# Changes of gut microbiota reflect the severity of major depressive disorder: a cross sectional study

**DOI:** 10.1038/s41398-023-02436-z

**Published:** 2023-04-28

**Authors:** Xi Hu, Yifan Li, Jing Wu, Hanping Zhang, Yu Huang, Xunmin Tan, Lu Wen, Xingyu Zhou, Peijun Xie, Oluwatayo Israel Olasunkanmi, Jingjing Zhou, Zuoli Sun, Min Liu, Guofu Zhang, Jian Yang, Peng Zheng, Peng Xie

**Affiliations:** 1 Department of Neurology, The First Affiliated Hospital of Chongqing Medical University, Chongqing, China; 2 NHC Key Laboratory of Diagnosis and Treatment on Brain Functional Diseases, The First Affiliated Hospital of Chongqing Medical University, Chongqing, China; 3 Beijing Key Laboratory of Mental Disorders, National Clinical Research Center for Mental Disorders & National Center for Mental Disorders, Beijing Anding Hospital, Capital Medical University, Beijing, China; 4 Advanced Innovation Center for Human Brain Protection, Capital Medical University, Beijing, China

**Keywords:** Depression, Diagnostic markers

## Abstract

Disturbed gut microbiota is a potential factor in the pathogenesis of major depressive disorder (MDD), yet whether gut microbiota dysbiosis is associated with the severity of MDD remains unclear. Here, we performed shotgun metagenomic profiling of cross-sectional stool samples from MDD (*n* = 138) and healthy controls (*n* = 155). The patients with MDD were divided into three groups according to Hamilton Depression Rating Scale 17 (HAMD-17), including mild (*n* = 24), moderate (*n* = 72) and severe (*n* = 42) individuals, respectively. We found that microbial diversity was closely related to the severity of MDD. Compared to HCs, the abundance of *Bacteroides* was significantly increased in both moderate and severe MDD, while *Ruminococcus* and *Eubacterium* depleted mainly in severe group. In addition, we identified 99 bacteria species specific to severity of depression. Furthermore, a panel of microbiota marker comprising of 37 bacteria species enabled to effectively distinguish MDD patients with different severity. Together, we identified different perturbation patterns of gut microbiota in mild-to-severe depression, and identified potential diagnostic and therapeutic targets.

## Introduction

Major depressive disorder (MDD) is the most common form of psychiatric and emotional disorder, affecting >350 million people [1]. Meanwhile, MDD has a high relapse rate [2] and causes an enormous social cost [3]. The baseline symptom severity of depression is one of the important factors influencing the treatment outcome [4, 5]. Mild to moderate depression can be treated conservatively without aggressive psychopharmacology [6], while severe depression may require antipsychotic, electroconvulsive, or other forms of therapy [4]. Clinically, the misdiagnosis of MDD will lead to ineffective antidepressant treatment, and even aggravate the disease. In addition, there is already a high risk of suicide or self-injury in severe cases of MDD. Therefore, it is of great clinical significance to identify new biomarkers for MDD patients with different severity, and this is crucial for early intervention.

Recently, growing evidence indicates that gut microbiota plays an essential role in the development and progression of mental disorders [7]. Alteration of gut microbiota was speculated to be the potential etiology of MDD, as it can affect the host’s brain function and behavior through the “gut-brain axis” [8]. Microbial biomarkers have been shown to be novel diagnostic and differential diagnostic tools, and help to identify new molecular therapeutic targets for diseases [9–13]. Generally, 16 S rRNA sequencing is mainly used to characterize the bacterial microbial composition, and explore the association between altered gut microbiota and various diseases [12, 13]. Previous studies have found that *Bacteroides* is the hub of perturbed gut microbiota in unipolar depression, while enriched *Prevotella* is the characteristic of bipolar depression [14]. In addition, patients with current active MDD (a-MDD) showed significantly increased in *Alistipes* and *Anaerostipes* as well as completely depleted *Dialister*, while mild symptoms (r-MDD) had higher abundance of *Bilophila* [15]. However, another study found *Bacteroidetes*, *Proteobacteria*, and *Actinobacteria* were strongly increased in a-MDD and r-MDD, whereas *Firmicutes* was significantly reduced [16]. These studies suggest that the gut microbiota may be different in MDD patients with different severity. However, due to the relative limited resolution of 16 S rRNA analysis, the identification level of bacteria can only be accurate to the genus level, and yield a small amount of information on species diversity. To make up for the gap of knowledge in this field, here, metagenome sequencing was used to characterize the gut microbial composition and function of MDD (*n* = 138), including mild (*n* = 24), moderate (*n* = 72) and severe patients (*n* = 42), and healthy controls (HCs, *n* = 155). Firstly, we sought to explore whether the whole microbial signature of MDD patients with different severity was significantly different from that in HCs. Next, we integrated the microbiota and related functional information through network analysis and correlated it with disease severity to further reveal how the signature of these disturbances changed as the disease worsened. Finally, we identified potential microbial markers related to severity of depression, and further tested their discriminative performance, thus making our findings a useful resource for the study of microbiome perturbations in depression.

## Methods

### Subject recruitment

The subjects included in this study were derived from our previous clinical cohort [17]. The study protocol was reviewed and approved by the Human Research and Ethics Committee of Beijing Anding Hospital (no. 2017-24), Capital Medical University (China). All recruited subjects signed a written informed consent. Each patient satisfied the MDD diagnostic criteria of the Diagnostic and Statistical Manual of Mental Disorders, 4th Edition (DSM-IV). The severity of MDD was staged with the HAMD-17 scale [18]. Depression severity stratification ranges as follows:[19] mild depression (score, 8–16); moderate depression (score, 17–23); and severe depression (score, ≥24). A total of 155 healthy controls (HCs; age, 29.13 ± 8.03; BMI, 22.38 ± 3.34) and 138 untreated MDD patients were recruited (age, 29.28 ± 7.10; BMI, 22.44 ± 3.41), including mild group (*n* = 24; age, 29.34 ± 7.64; BMI, 22.56 ± 3.99), moderate group (*n* = 72; age, 29.99 ± 7.31; BMI, 22.69 ± 3.21), severe group (*n* = 42; age, 28.08 ± 6.36; BMI, 21.93 ± 3.40). All patients provided written informed consent to participate. Patients that were excluded from this study were those who (1) had bipolar disorders, schizophrenic, schizoaffective, or other Axis I psychiatric disorders; (2) had the serious chronic somatic disease (diabetes, cardiovascular disease, thyroid disease, cancer, etc.); (3) alcohol and substance abuse, acute intoxication; (4) were pregnant or breastfeeding; (5) changed diet habit or used antibiotic within one month before sampling.

### Metagenomic analysis of fecal samples

#### Fecal DNA extraction

All samples were collected from the clinical center. Briefly, fresh stool samples were collected and contained in sterile tubes in the morning (7–10 am) and stored at 4 °C, then transferred to a −80 °C refrigerator for subsequent processing within 6 h. According to the manufacturer’s instruction, we extracted the whole genomic DNA from fecal samples with the E.Z.N.A. Soil DNA Kit (Omega Bio-Tek, Norcross, GA, USA). Determination of the extracted DNA’s concentration and purity was performed on the TBS-380 and NanoDrop2000 separately. Then, we checked the quality on 1% agarose gel. DNA was fragmented randomly to an average size of about 300 bp by Covaris M220. Construction of paired-end library was accomplished by NEXTFLEX Rapid DNA-Seq (Bio-Scientific, Austin, TX, USA). Library was subjected to paired-end sequencing on Illumina NovaSeq (Illumina Inc., San Diego, CA, USA). To avoid batch effect, all samples were assayed in the same batch.

#### Quality control of raw sequences and data analysis

Low-quality sequences (sequences that were shorter than 50 bp or homopolymers that were longer than 10 bp or contained ambiguous base calls) in raw FASTQ files were filtered by Sickle (https://github.com/najoshi/sickle). Metagenomic data were aligned to the human genome using Burrows-Wheeler Aligner (http://bio-bwa.sourceforge.net), and the host genes were removed. Clean data were assembled to contigs by MEGAHIT, and the contigs with a minimum length of 300 bp were kept. Metagene was used to predict open reading frames from each assembly contigs [20]. All genes predicted to have 95% sequence identity were clustered using CD-HIT [21]. After completing the above procedures, reads were mapped to the representative sequences using SOAPaligner.

#### Metagenome data analysis

The gene set was annotated for bacteria based on the NCBI database using Diamond (version 0.8.35). For assessing the gut microbiota species of MDD patients, each gene was assigned to the highest-scoring taxonomy based on a unified database. Non-redundant gene set was aligned against the KEGG database with an e value cutoff of 1 × 10^−5^ [22], and abundance of the KO was calculated from the sum of the abundances of the genes corresponding to the KO. The gene expression value of gene set which used for species and function annotation were all based on the Reads Per Kilobase Million (RPKM). The α-diversity indexes were calculated by past 4.0. The α-diversity analysis was performed based on 4 indexes (Dominance, Simpson, Shannon and Evenness). Principal coordinate analysis (PCoA) based on Bray-Curtis distance was used to evaluate the overall difference of bacterial communities among HCs and MDD subgroups [23]. Permutational multivariate analysis of variance (PERMANOVA) was used to test the overall and pairwise group differences. PCoA analysis and PERMANOVA were based on the relative abundance of species. Samples were clustering into enterotypes in genus level by Dirichlet multinomial mixtures (DMM) approach as previous study described [24]. Optimal number of clusters was determined by Calinski-Harabasz index. Enterotypes analysis was based on the abundance of genus.

#### Combined biomarker for MDD subgroups

High relative abundance bacteria were selected for following analysis at species level (prevalence >20%, average relative abundance >0.01%), then unclassified species was excluded. Linear discriminant analysis effect size (LEfSe) analysis was used to identify the differentially enriched bacteria and KOs among HCs and 3 MDD subgroups (LDA score >2.5, https://huttenhower.sph.harvard.edu/galaxy/). The diagnostic performance was quantified by Random Forest classifier and tested by 5-fold cross-validation. Receiver operating characteristic (ROC) curve was plotted to estimate the diagnostic efficacy.

#### Construction of co-occurrence network of gut bacteria

Based on abundance data of metagenome, Sparse correlations for compositional data (SparCC) algorithm was used to calculate the correlations between all the differentially enriched bacteria and KOs which defined by LEfSe analysis (*p* < 0.05, http://mem.rcees.ac.cn:8081) [25]. The result was visualized by Cytoscape (version 3.9.0) and Graphpad Prime 8.

#### Statistical analysis

Statistical analysis was performed using SPSS (version 22.0). Continuous variables were analyzed by one-way ANOVA test (mean±SD) followed by LSD′s multiple comparison or non-parametric factorial Kruskal–Wallis sum-rank test (mean±SEM), *p* values of pairwise comparisons of the Kruskal–Wallis sum-rank test were corrected by Holm-Bonferroni method. Categorical variables were performed by chi-square test. Statistical significance level was set at *p* < 0.05.

## Results

### Clinical characteristics of the subjects

In this study, we included in a total of 155 HCs and 138 MDD patients. All MDD patients were treatment-naive, and there was no significant difference in gender (*p* = 0.71; Chi-square test), age (*p* = 0.62; one-way ANOVA), and body mass index (BMI; *p* = 0.71; one-way ANOVA) among HCs and 3 MDD subgroups (Table S1).

### Altered gut microbiota among HCs and MDD subgroups

**Fig. 1:**
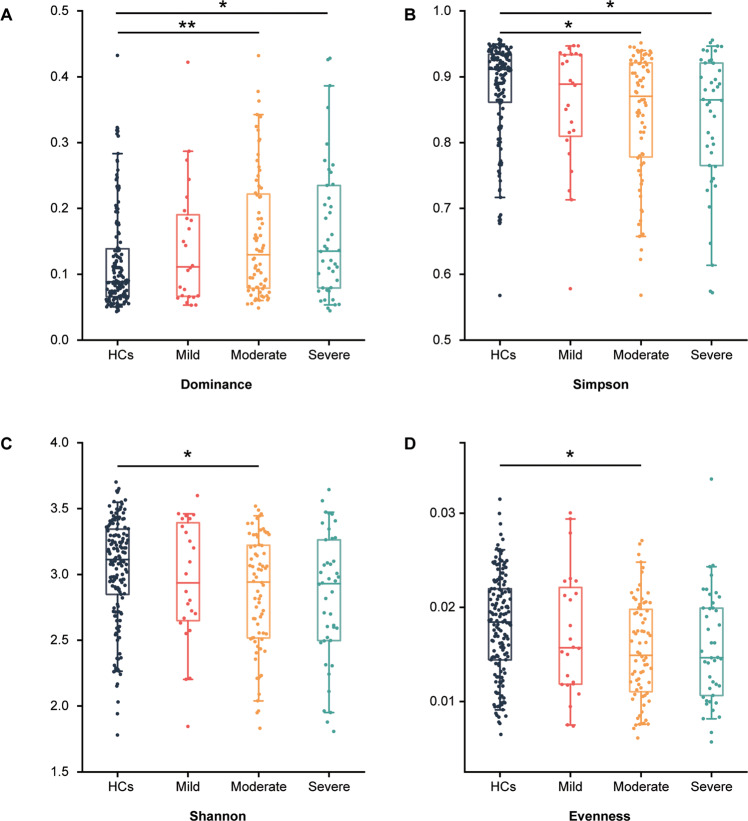
The α-diversity index analysis among HCs and MDD subgroups. **A**–**D** The box plots showing α-phylogenetic diversity analysis results, the dominance index increased in moderate and severe groups relative to HCs while simpson was decreased, while there was no significant difference between mild and HCs. In moderate group, the shannon and evenness indexes were significantly lower than that in HCs. (HCs, *n* = 155; mild, *n* = 24; moderate, *n* = 72; severe, *n* = 42; ^*^p < 0.05; ^**^p < 0.01; Kruskal–Wallis rank sum test).

**Fig. 2:**
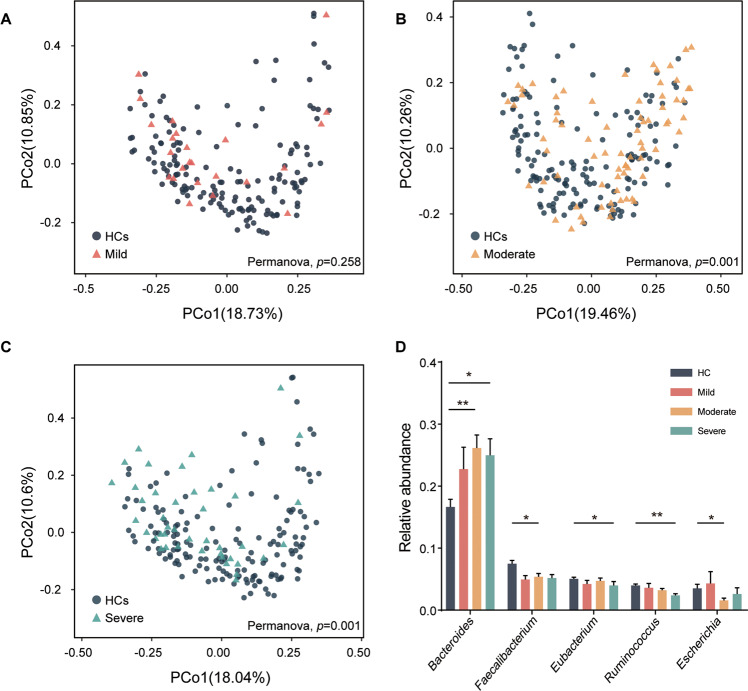
Gut microbial characteristics among HCs and MDD subgroups. **A**–**C** Principal co-ordinates analysis (PCoA) was conducted based on species level and Bray-Curtis distance. PERMANOVA test showed that the general characteristics of microbiota in moderate and severe groups were significantly different from HCs, while there was no significant difference between mild group and HCs. In addition, we did not find the separated characteristics of microbiota by comparing disease subgroups (see Fig. S2). **D** The bar plot showed the differentially enriched genus in the most top 10 high relative abundance genus. *Bacteroides* was significantly increased in moderate and severe group, while the reduction of 2 genera (*Faecalibacterium* and *Escherichia)* in moderate group and 2 genera (*Eubacterium* and *Ruminococcus)* in severe group, respectively. (^*^p < 0.05; ^**^p < 0.01; Kruskal–Wallis rank sum test).

Initially, the α-diversity was compared among HCs and 3 MDD subgroups. Consequently, we found that the simpson index of moderate and severe groups was significantly decreased, while there was no significant difference between the mild group and the others (Fig. 1A, B). In addition, the shannon and evenness indexes were decreased in moderate group (Fig. 1C, D). These findings suggested that the community richness and diversity were associated with MDD severity. To explore the difference in microbial signatures among HCs and 3 subgroups, PCoA was performed. We found that the whole microbial compositions of the moderate and severe groups were different from those of the HCs; while the compositions of 3 MDD subgroups were not different from each other (Fig. 2A–C, Fig. S2).

Next, we investigated the high relative abundance (top 10) bacteria of HCs and the 3 MDD subgroups at family and genus levels. Overall, at the family level, *Bacteroidaceae, Lachnospiraceae, Ruminococcaceae* and *Prevotellaceae* were the major high abundance bacterial taxa (Fig. S1A). At genus level, *Bacteroides, Faecalibacterium, Blautia, Prevotellaceae* were the major high abundance bacterial taxa (Fig. S1B). Here, we found that, compared to HCs, *Bacteroides* were remarkably enriched in moderate and severe groups, *Faecalibacterium* and *Escherichia* were decreased in moderate group, while *Ruminococcus* and *Eubacterium* were decreased only in severe group (Fig. 2D). Next, we explored the distribution of different enterotype in HCs and 3 MDD subgroups. Based on Dirichlet multinomial mixtures (DMM) approach, we observed 5 enterotypes. Except for the classical enterotypes (*Bacteroides*; *Prevotella*) [26–28], we distinguished 2 new enterotype which dominated by *Blautia* and *Faecalibacterium*. *Faecalibacterium* (31.6%) were significantly more abundant in HCs, whereas 2 types of *Bacteroides* (*Bac, Bac2*) were more abundant in 3 MDD subgroups (mild: 33.3%, moderate: 36.1%, severe: 31.0%). 3 MDD subgroups shared a similar trend that *Bac* and *Bac 2* were the major enterotypes in microbiota (Fig. S1C).

**Fig. 3:**
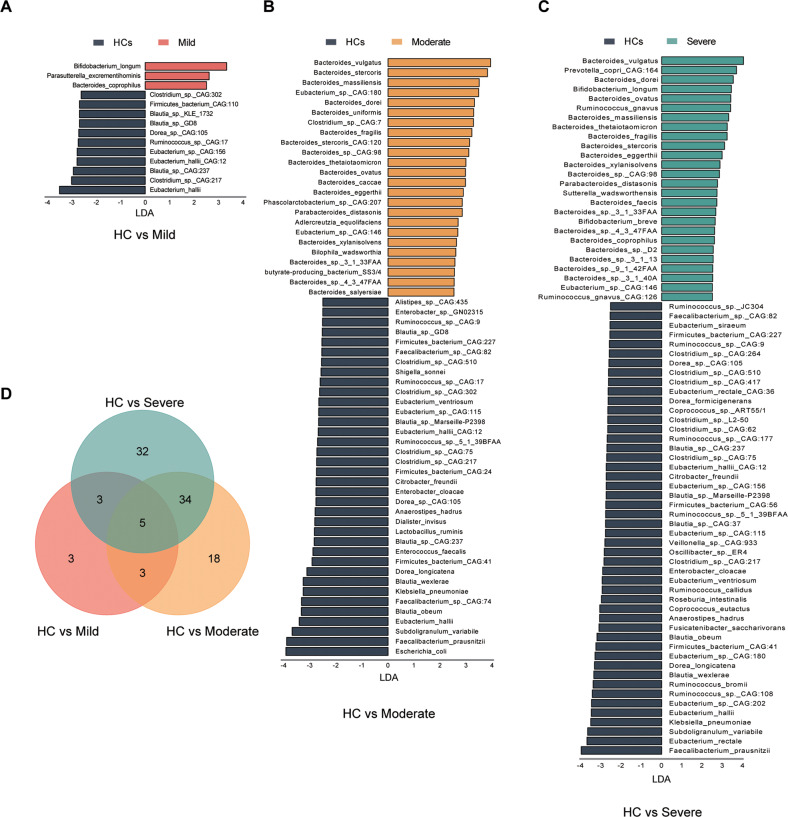
Differentially enriched bacteria species of HCs and the 3 MDD subgroups. LDA Effect Size (LEfSe) analysis was performed to identify the bacteria that were differentially enriched in HCs and the 3 MDD subgroups in pairs (LDA > 2.5, *p* < 0.05). **A**–**C** Bar plot displayed the differentially enriched species between HC and MDD subgroups. **D** Venn diagram showed that moderate and severe group shared 39 differentially enriched bacteria, while only 5 bacteria were shared in the 3 MDD subgroups.

To further gain insight into the alterations of gut microbiota and the consequence of the profound bacteria derangement on metabolic function, LDA Effect Size (LEfSe) analysis was performed pairwise on bacteria between HCs and MDD subgroups (LDA > 2.5, *p* < 0.05). Three groups of bacteria were identified through three pairwise comparisons of LEfSe analysis (Table S2–4). Compared with HCs, mild group had less differentially enriched bacteria at the set threshold. We found 14 differentially enriched bacteria in mild group, 60 in moderate group, and 74 in severe group (Fig. 3A–C). Those decreased gut bacteria of the mild group mainly belong to *Blautia* (3 species) and *Eubacterium* (3 species). *Blautia* (5 species), *Clostridium* (4 species) and *Eubacterium* (4 species) were decreased in moderate group, while *Bacteroides* (16 species) were remarkable increased in moderate group. Consistently, *Blautia* (5 species), *Clostridium* (7 species) and *Eubacterium* (8 species) were also decreased in severe group, *Ruminococcus* (7 species) were decreased too. The increased bacteria remained *Bacteroides* (18 species) in severe group (Table S2–4). Venn diagram showed that there were 99 differentially enriched bacteria among HCs and 3 MDD subgroups. Three bacteria were exclusive in mild group, 18 in moderate group and 32 in severe group (Fig. 3D). Interestingly, we found that 4 of the 18 unique bacteria of moderate group belonged to *Bacteroides*. Five of the 32 unique bacteria of severe belonged to *Bacteroides*, 4 belonged to *Clostridium* and 6 belonged to *Ruminococcus*. Likewise, we found 3 differentially enriched KOs in moderate group and 5 in severe group (K03088, K21572, K07133, Table S5-6). There were 2 KOs that were exclusive in severe group (K01190, K12373, Table S6). K01190 encodes lactase to uptake and hydrolyze lactose [29]. K12373 encodes hexosaminidase involve in glycosphingolipid biosynthesis, and the lack of hexosaminidase could cause gangliosidosis [30].

### Concordance of microbial variation in patients with moderate and severe MDD

**Fig. 4:**
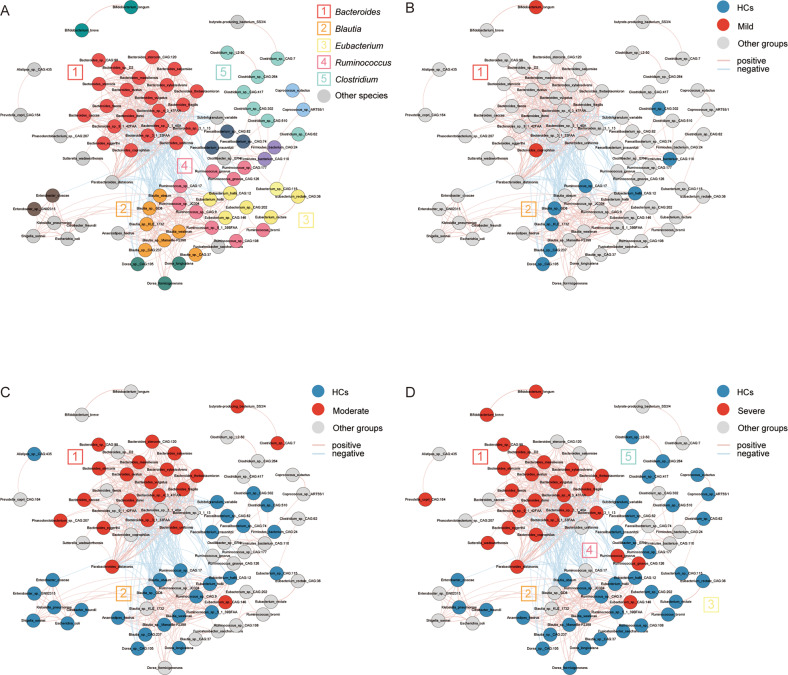
The co-occurrence network constructed from the relative abundance of differentially enriched bacteria species among HCs and MDD subgroups. The network was mapped based on the result (*p* < 0.05, r^2^ > 0.25). **A** Correlation among abundances of all differentially enriched bacteria were analyzed with SparCC algorithm, clusters were assigned a particular color and main clusters had a corresponding number. **B**, **D** Species enriched in MDD subgroups were highlighted in red and depleted ones were in blue. Pink lines represented positive correlations and blue lines means negative correlations. **B** Bacteria altered little in mild group, some scattered species were depleted in mild groups associated with each other weakly and had no correlation with the only enriched *Bacteroides* in cluster 1. **C** Bacteria enriched in moderate groups were almost all belong to *Bacteroides* in cluster 1, the major depleted genus was *Blutia* in cluster 2, which have a significantly negative association with *Bacteroides*. **D** Gut microbiota of severe group appeared fierce disturbance featured with markable increase of *Bacteroides* in cluster 1. *Ruminococcus*, *Blautia*, *Eubacterium* and *Clostridium* (cluster 2, 3, 4, 5) were obviously decreased as a group. There were complex positive correlations within cluster 2, 3, 4 and a significantly negative correlation between the decreased group and *Bacteroides*. It revealed that there was a potential antagonistic relationship between the decreased group and *Bacteroides*.

In order to explore the potential correlations among the differentially enriched microbiota, SparCC correlation analysis was performed to assess the co-occurrence relationship between the differential enriched bacteria. Based on these results, we constructed a co-occurrence network (*p* < 0.05, r^2^ > 0.25). In genus level, *Bacteroides* enriched markedly in every MDD subgroup (Fig. 4A–D). Notably, we found that the dominant depleted bacteria varied in MDD subgroups. The characteristic microbiota of the severe group were *Ruminococcus* (6/9), *Eubacterium* (6/7), *Blutia* (5/7) and *Clostridium* (5/7) (Fig. 4D), while that of moderate group were *Blutia* (5/7) (Fig. 4C). The enriched bacteria *Bacteroides* showed a strong negative correlation with the depleted bacteria, particularly the *Blautia*, *Ruminococcus*, *Eubacterium* (Fig. 4A). This phenomenon was indicative of a competitive antagonistic relationship among those bacteria. In addition, we also explored the correlation among those changed bacteria and KOs. The differentially enriched KOs and species that highly correlated with either KOs were reserved to construct the correlation-based heatmap (r^2^ > 0.25). Overall, *Bacteroides* were positively correlated with KOs and *Blautia, Eubacterium, Ruminococcus* were negatively correlated with KOs (Fig. S3A). Obviously, K12373 and K21572 were highly positively correlated with 21 *Bacteroides* while negatively correlated with *Blautia* and *Ruminococcus* (Fig. S3B). Compared with K12373, there were 3 more species of *Blautia* associated with K21572 (Fig. S3B).

### Identification of faecal bacteria species as potential biomarker for different severity of MDD

**Fig. 5:**
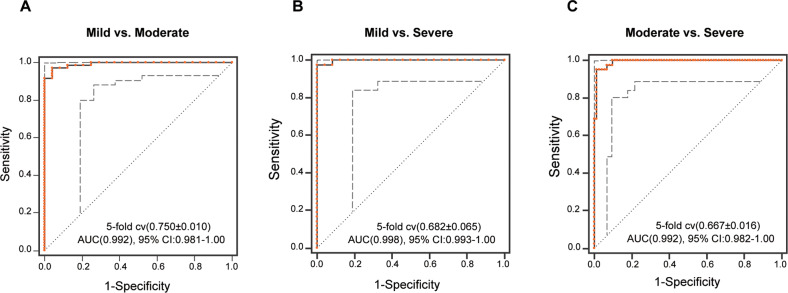
Diagnosing MDD subgroups from gut microbiome features. **A**–**C** Based on the importance value of random forest analysis (>2%) between MDD subgroups and HCs, 37 species was identified (see Table S2–4 for importance value). This microbial panel enabled the differentiation between any 2 subgroups with high diagnostic accuracy (AUC, 0.992–0.998).

The differentially enriched bacteria of HCs and 3 subgroups were trained separately by random forest classifier (RF) for binary classification. According to Gini importance, 37 bacteria were screened out from 3 pairs of differentially enriched bacteria to construct the biomarker panel for discriminating different groups (Gini importance>0.02, Table S7). The diagnostic performance of paired identification was evaluated by receiver operating characteristic (ROC) curves and quantified by the area under the curve (AUC). AUC of the bacterial biomarker ranged from 0.992 to 0.998 (mild versus moderate, AUC = 0.992; mild versus severe, AUC = 0.998; moderate versus severe, AUC = 0.992; Fig. 5A–C). It reveals that the diagnostic performance of biomarkers was excellent.

### Sex-specific of altered gut microbiota

Considering of the gender differences in the prevalence and clinical manifestations of depression, we explored the gender bias of gut microbiota characteristic. In both female and male groups, species diversity declined significantly in MDD subgroups relative to HCs (Fig. S4A, B). Shannon and simpson indexes were decreased in MDD subgroups. MDD subgroups were remarkably separated from HCs in both female and male groups (Fig. S5A, B). The changes in composition were also similar in two genders. Compared the percentage of top 10 bacteria in samples, we found the *Bacteroidaceae* increased while *Ruminococcaceae* decreased at family level in MDD subgroups, *Bacteroides* increased while *Faecalibacterium* decreased at genus level in MDD subgroups (Fig. S6).

LEfSe analysis was performed to screen out the differentially enriched bacteria (LDA > 2.5, *p* < 0.05). Ultimately, we identified 38 species differentially enriched bacteria in female group (Fig. S7A, Table S8), most of them belonged to *Bacteroides* (11 species), *Eubacterium* (7 species), *Clostridium* (4 species) and *Blautia* (4 species); and 68 species differential enriched bacteria in male group (Fig.S7B, Table S9), most of them belonged to *Bacteroides* (17 species), *Eubacterium* (6 species), *Clostridium* (4 species), *Blautia* (6 species) and Ruminococcus (4 species).

Identically, we constructed co-occurrence networks based on SparCC analysis of differentially enriched bacteria (*p* < 0.05, r^2^ > 0.25). In female group, there were 3 hub clusters, the *Bacteroides* showed a negative correlation with *Eubacterium* and *Blautia* (Fig. S8A). In the male group, a positively correlated network was found among *Blautia*, *Ruminococcus, Eubacterium* and *Coprococcus*, while they showed negative correlations with *Bacteroides* (Fig. S8B).

## Discussion

The link between changes in the gut microbiome and MDD has been supported by several studies [14, 17]. Here, we showed that these changes could reflect the severity of MDD. In this study, we found that the gut microbiota of moderate and severe MDD patients were characterized by the enrichment of *Bacteroidetes*, while *Ruminococcus* and *Eubacterium* were depleted in the severe patients. Consistently, the major enterotype of HCs was *Faecalibacterium* while which of MDD subgroups were *Bac* and *Bac 2*. In addition, we also identified a microbial marker panel which is capable of distinguishing MDD patients with different severity.

It is generally believed that high diversity of gut microbiome is a sign of healthy status [31, 32]. Some studies have found that the gut microbial diversity reduced in depression, bipolar disorder and schizophrenia [33]. In addition, reduced diversity has been associated with disease severity and higher risk of death in patients with diseases such as bronchiectasis [34], cystic fibrosis [35] and ulcerative colitis [36]. Consistently, we found that the simpson index decreased only in the moderate and severe MDD groups at the genus level. In addition, the Venn diagram showed that the number of shared species between moderate and severe is higher than that of other groups. Bar plot showed that *Bacteroides* were significantly enriched in moderate and severe subgroups, while there was no change in the mild group. These findings suggested that the gut microbial composition remains relatively stable during the early stages of MDD; but with increase in disease severity, the disturbances of gut microbiota become inevitable. Furthermore, based on the LDA effect size analysis, the differentially expressed microbiota in the MDD subgroups were identified. We constructed the co-occurrence network of perturbed microbiota of MDD with mild to severe. We found that the depletion of *Blautia* and *Eubacterium* were common features of MDD patients; enriched *Bacteroides* were characteristic of moderate and severe MDD. Consistently, our previous study proved that increase of *Bacteroides* was a signature of MDD [17]. *Bacteroides* was reported involved in immune system maturation, tumor formation and activation of autoimmune disease by affecting T cell’s function and promote cognitive impairment disease pathologies through activating microglia [37–40]. *Bacteroides faecis* helps maintaining the epithelial barrier integrity and increasing the gut IgA level to reduce inflammatory bowel disease [41, 42]. Because of related closely, *Bacteroides dorei* and *Bacteroides vulgatus* are often researched as a group, they were been reported playing an important role in brown adipose tissue metabolism and suppressing proinflammatory immune responses [43, 44]. Similarly, researched revealed that *Bacteroides uniformis* and *Bacteroides eggerthii* were involved in pathological mechanism of obesity, metabolism, and colitis [45–47]. In some studies, *Blautia* have been reported as potential probiotics that can induce anti-inflammatory peripheral immune response, alleviate obesity-related disease and regulate metabolism through cross-feeding with other bacteria [48–50]. Also, it has been reported that *Eubacterium* can produce short-chain fatty acids, which play an important role in regulating cell metabolism, immune and endocrine response [51, 52]. In addition, unlike mild and moderate patients, the highly concentrated clusters in severe MDD were dominated by decreased 7 *Ruminococcus*, 8 *Eubacterium*, 5 *Clostridium* and 7 *Clostridium*. Depletion of these potential probiotics may contribute to the development of depression. Compared with patients with mild and moderate depression, the severe individuals need more active physical and drug therapy. Therefore, combining a probiotic intervention strategy with conventional treatment will be helpful in promoting the improvement of both disease recovery and quality of life. In addition, we found that K21572 (*susD*) and K12373 (*HEXA*) may be key node connecting *Blautia* and *Bacteroides*, showing completely opposite correlation with these two microbial clusters. *susD* is an outer membrane protein. It is the main starch binding protein on the surface of *Bacteroides*, and can effectively use polysaccharides as a source of carbon and energy [53]. Further work characterizing the interaction between *Blautia* and *Bacteroides* could elucidate its role in MDD severity. K12373 (*HEXA*) is a hexose kinas, play an important role in sugar metabolism in *Bacteroides fragilis* [54]. Likewise, K03088 (*rpoE*) is an important part of transcription activation factor that binds to RNA polymerase complex to regulate gene expression in bacteria [55]. In sight of the positive association of K03088 and *Bacteroides*, the increased expression of K03088 might indicate the function enhancement of *Bacteroides*.

Motivated by the results that showed that there were alterations in the gut microbiota in moderate and severe MDD, we constructed a random forest model with 37 bacteria species. The AUC value of the classification of MDD subgroups were 0.992 to 0.998; suggesting a high diagnostic value. Overall, this finding provided evidence that gut microbiota-targeted biomarkers may become potential non-invasive tools for MDD stratification.

Additionally, we initially explored the gender bias in the structure of gut microbiota. The prevalence of MDD in women was twice of in men [56, 57], we wondered if there is a potential relationship between gender preference of disease and gut microbiota. Generally, we found that the changes of microbiota were similar in female and male. Previous study declared that *Bacteroides* and *Prevotella* had higher abundance in male [58]. In our study, male group owned more differentially enriched bacteria and most of them were belonged *Bacteroides*. Further research was required to figure out if this was related to gender differences in MDD. In order to obtain consolidation evidence, it is necessary to further expand the study cohort to determine the gender bias of gut microbiota in MDD.

The limitations of this study are: (i) The sample size of the three MDD subgroups is relatively small, and the samples were collected from a clinical center, therefore regional variations cannot be ruled out; (ii) All patients were not under medication; therefore, follow-up studies are required to explore whether this biomarker panel can be used to monitor treatment response; (iii) Given that fecal bacteria transplantation could transfer depressive phenotypes from humans to mice, it will be important to determine the correlation between the disturbance of gut microbiota and disease severity in animal models, and uncover the underlying mechanisms.

Taken together, we analyzed and found the unique and common alterations in gut microbiota across different disease severities. Microbiota may affect physiological functions through mutual synergy or antagonism, and the status may shift from balance to imbalance as symptoms get worse. Furthermore, we identified a novel combined biomarker that could discriminate different severity subgroups with high accuracy. In conclusion, our study provides a new direction for understanding the progression of MDD, and a potential promising strategy for developing a novel method for objectively assessing the severity of MDD.

## Supplementary information

Supplemental tables

Supplementary materials

Storms checklist

aj-checklist

supplementary figure 1

supplementary figure 2

supplementary figure 3

supplementary figure 4

supplementary figure 5

supplementary figure 6

supplementary figure 7

supplementary figure 8

## Data Availability

The metagenomic sequencing data were deposited in the China National GeneBank DataBase (CNGBdb) (https://db.cngb.org/; project ID: CNP0001162).

## Code availability

All the source codes are available at https://github.com/mokmoksunshine/changes_of_gut_microbiota_severity.
