# Supplementary material for: Changes of gut microbiota reflect the severity of major depressive disorder: a cross sectional study
Source: Transl Psychiatry. 2023 Apr 28;13:137. doi: 10.1038/s41398-023-02436-z (PMC10147706; doi:10.1038/s41398-023-02436-z)
Supplement: Supplementary file 1 — Supplemental tables [file 41398_2023_2436_MOESM1_ESM.docx]

**Supplementary Tables**

**Supplementary Table 1. Demographic information of the subjects.**

**Supplementary Table 2. Discriminatory bacteria species between HCs and mild groups.**

**Supplementary Table 3. Discriminatory bacteria species between HCs and moderate groups.**

**Supplementary Table 4. Discriminatory bacteria species between HCs and severe groups.**

**Supplementary Table 5. Discriminatory KOs between HCs and moderate group.**

**Supplementary Table 6. Discriminatory KOs between HCs and severe group.**

**Supplementary Table 7. Potential biomarker for identifying different severity of MDD**

**Supplementary Table 8. Discriminatory bacteria between HCs and MDD in female samples**

**Supplementary Table 9. Discriminatory bacteria between HCs and MDD in male samples**

**[Tables below]**

**Supplementary Table1.****Demographic information of the subjects.**

| **Variables** | **Mild**  **(n=24)** | **Moderate**  **(n=72)** | **Severe**  **(n=42)** | **HCs**  **(n=155)** | ***p*** |
| --- | --- | --- | --- | --- | --- |
| Age (yr, mean± SD) | 29.34±7.64 | 29.99±7.31 | 28.02±6.36 | 29.13±8.03 | 0.62^a^ |
| BMI (mean± SD) | 22.56±3.99 | 22.69±3.21 | 21.93±3.40 | 22.38±3.34 | 0.71^a^ |
| Gender, male% (n) | 33.33%(8) | 55.56%(40) | 33.33%(14) | 41.29%(64) | 0.71^b^ |

**HCs: healthy controls;**

**^a^ One-way anova test;**

**^b^ Chi-square test;**

**Supplementary Table 2.** **Discriminatory bacteria species between HCs and mild group.**

| Genus | Species | Relative abundance | | | | Enrichment | LDA | *p* value | Importance |
| --- | --- | --- | --- | --- | --- | --- | --- | --- | --- |
|  |  | HCs | | mild | |  |  |  |  |
|  |  | mean | sem | mean | sem |  |  |  |  |
| *Parasutterella* | *Parasutterella_excrementihominis* | 6.37E-04 | 1.03E-04 | 1.48E-03 | 3.80E-04 | Mild | 2.62 | 2.60E-02 | 0.11 |
| *Bacteroides* | *Bacteroides_coprophilus* | 1.69E-03 | 3.43E-04 | 2.09E-03 | 4.79E-04 | Mild | 2.51 | 3.84E-02 | 0.09 |
| *Ruminococcus* | *Ruminococcus_sp._CAG:17* | 1.44E-03 | 2.78E-04 | 3.96E-04 | 5.19E-05 | HC | 2.74 | 2.09E-03 | 0.09 |
| *Clostridium* | *Clostridium_sp._CAG:302* | 1.21E-03 | 3.48E-04 | 5.58E-04 | 3.04E-04 | HC | 2.62 | 2.49E-02 | 0.09 |
| *Bifidobacterium* | *Bifidobacterium_longum* | 5.61E-03 | 6.72E-04 | 9.42E-03 | 3.14E-03 | Mild | 3.33 | 1.72E-02 | 0.08 |
| *Eubacterium* | *Eubacterium_sp._CAG:156* | 1.42E-03 | 3.26E-04 | 3.07E-04 | 8.77E-05 | HC | 2.78 | 8.67E-03 | 0.08 |
| *Eubacterium* | *Eubacterium_hallii* | 9.44E-03 | 1.06E-03 | 3.46E-03 | 9.32E-04 | HC | 3.50 | 1.06E-02 | 0.07 |
| *Eubacterium* | *Eubacterium_hallii_CAG:12* | 1.64E-03 | 1.94E-04 | 5.65E-04 | 1.72E-04 | HC | 2.79 | 1.08E-02 | 0.07 |
| *Dorea* | *Dorea_sp._CAG:105* | 1.76E-03 | 3.39E-04 | 6.46E-04 | 4.18E-04 | HC | 2.71 | 4.39E-03 | 0.07 |
| *Blautia* | *Blautia_sp._CAG:237* | 2.27E-03 | 9.41E-04 | 6.21E-04 | 1.97E-04 | HC | 2.94 | 2.28E-02 | 0.06 |
| *Blautia* | *Blautia_sp._KLE_1732* | 1.78E-03 | 1.78E-04 | 8.74E-04 | 1.46E-04 | HC | 2.69 | 2.60E-02 | 0.05 |
| *Blautia* | *Blautia_sp._GD8* | 1.79E-03 | 1.71E-04 | 8.87E-04 | 1.23E-04 | HC | 2.69 | 9.93E-03 | 0.05 |
| *unclassified_p_Firmicutes* | *Firmicutes_bacterium_CAG:110* | 1.28E-03 | 1.97E-04 | 4.59E-04 | 1.44E-04 | HC | 2.69 | 3.25E-02 | 0.05 |
| *Clostridium* | *Clostridium_sp._CAG:217* | 2.58E-03 | 4.79E-04 | 5.92E-04 | 2.97E-04 | HC | 3.01 | 3.50E-02 | 0.04 |

**Supplementary Table 3. Discriminatory bacteria species between HCs and moderate group.**

| Genus | Species | Relative abundance | | | | Enrichment | LDA | *p* value | Importance |
| --- | --- | --- | --- | --- | --- | --- | --- | --- | --- |
|  |  | HCs | | Moderate | |  |  |  |  |
|  |  | mean | sem | mean | sem |  |  |  |  |
| *Klebsiella* | *Klebsiella_pneumoniae* | 7.10E-03 | 2.23E-03 | 4.39E-03 | 2.04E-03 | HC | 3.26 | 4.80E-06 | 0.07 |
| *Enterobacter* | *Enterobacter_cloacae* | 1.65E-03 | 6.76E-04 | 6.79E-04 | 3.39E-04 | HC | 2.77 | 5.26E-05 | 0.04 |
| *Bacteroides* | *Bacteroides_stercoris* | 5.81E-03 | 1.04E-03 | 1.93E-02 | 3.09E-03 | Moderate | 3.82 | 2.24E-05 | 0.04 |
| *Shigella* | *Shigella_sonnei* | 1.25E-03 | 2.54E-04 | 5.21E-04 | 1.34E-04 | HC | 2.57 | 2.47E-03 | 0.03 |
| *Bacteroides* | *Bacteroides_stercoris_CAG:120* | 1.10E-03 | 2.15E-04 | 3.84E-03 | 6.54E-04 | Moderate | 3.13 | 4.62E-05 | 0.03 |
| *Eubacterium* | *Eubacterium_sp._CAG:146* | 1.92E-03 | 2.58E-04 | 2.52E-03 | 6.82E-04 | Moderate | 2.68 | 1.69E-03 | 0.03 |
| *unclassified_p_Firmicutes* | *Firmicutes_bacterium_CAG:41* | 5.95E-03 | 7.58E-04 | 4.19E-03 | 7.97E-04 | HC | 2.92 | 3.03E-03 | 0.03 |
| *Bilophila* | *Bilophila_wadsworthia* | 9.05E-04 | 1.34E-04 | 1.71E-03 | 3.91E-04 | Moderate | 2.60 | 2.18E-03 | 0.03 |
| *Bacteroides* | *Bacteroides_sp._CAG:98* | 8.64E-04 | 2.16E-04 | 3.50E-03 | 9.24E-04 | Moderate | 3.10 | 6.11E-04 | 0.03 |
| *unclassified_o_Clostridiales* | *butyrate-producing_bacterium_SS3/4* | 1.22E-03 | 8.25E-05 | 1.87E-03 | 2.14E-04 | Moderate | 2.55 | 7.51E-03 | 0.02 |
| *Enterobacter* | *Enterobacter_sp._GN02315* | 2.57E-04 | 2.51E-04 | 1.18E-05 | 9.34E-06 | HC | 2.51 | 4.98E-09 | 0.02 |
| *Clostridium* | *Clostridium_sp._CAG:302* | 1.21E-03 | 3.48E-04 | 4.99E-04 | 2.41E-04 | HC | 2.62 | 1.63E-03 | 0.02 |
| *Bacteroides* | *Bacteroides_massiliensis* | 3.03E-03 | 5.36E-04 | 9.67E-03 | 2.18E-03 | Moderate | 3.50 | 1.41E-04 | 0.02 |
| *unclassified_p_Firmicutes* | *Firmicutes_bacterium_CAG:227* | 1.35E-03 | 2.58E-04 | 6.12E-04 | 1.08E-04 | HC | 2.54 | 9.20E-04 | 0.02 |
| *Clostridium* | *Clostridium_sp._CAG:75* | 1.39E-03 | 5.70E-04 | 4.03E-04 | 1.26E-04 | HC | 2.73 | 4.53E-02 | 0.02 |
| *Eubacterium* | *Eubacterium_hallii* | 9.44E-03 | 1.06E-03 | 4.19E-03 | 7.57E-04 | HC | 3.41 | 2.29E-05 | 0.02 |
| *Escherichia* | *Escherichia_coli* | 2.81E-02 | 5.49E-03 | 1.17E-02 | 2.96E-03 | HC | 3.92 | 1.84E-03 | 0.02 |
| *Citrobacter* | *Citrobacter_freundii* | 9.81E-04 | 6.09E-04 | 9.12E-05 | 2.14E-05 | HC | 2.76 | 3.54E-04 | 0.02 |
| *Alistipes* | *Alistipes_sp._CAG:435* | 8.58E-04 | 6.58E-04 | 1.56E-04 | 1.05E-04 | HC | 2.50 | 3.26E-02 | 0.02 |
| *Clostridium* | *Clostridium_sp._CAG:7* | 3.82E-03 | 4.59E-04 | 8.03E-03 | 1.62E-03 | Moderate | 3.28 | 2.69E-02 | 0.02 |
| *Dorea* | *Dorea_sp._CAG:105* | 1.76E-03 | 3.39E-04 | 5.43E-04 | 1.48E-04 | HC | 2.77 | 1.60E-05 | 0.02 |
| *Parabacteroides* | *Parabacteroides_distasonis* | 2.57E-03 | 2.25E-04 | 4.15E-03 | 4.13E-04 | Moderate | 2.85 | 6.69E-05 | 0.02 |
| *Bacteroides* | *Bacteroides_sp._4_3_47FAA* | 7.96E-04 | 8.18E-05 | 1.51E-03 | 1.76E-04 | Moderate | 2.54 | 2.36E-05 | 0.02 |
| *Eubacterium* | *Eubacterium_sp._CAG:180* | 7.71E-03 | 1.63E-03 | 1.11E-02 | 3.99E-03 | Moderate | 3.48 | 2.45E-02 | 0.01 |
| *Eubacterium* | *Eubacterium_hallii_CAG:12* | 1.64E-03 | 1.94E-04 | 6.75E-04 | 1.40E-04 | HC | 2.68 | 3.17E-05 | 0.01 |
| *Eubacterium* | *Eubacterium_ventriosum* | 3.35E-03 | 3.31E-04 | 2.60E-03 | 4.98E-04 | HC | 2.66 | 2.30E-02 | 0.01 |
| *Dialister* | *Dialister_invisus* | 1.79E-03 | 6.68E-04 | 3.14E-04 | 1.50E-04 | HC | 2.81 | 3.87E-02 | 0.01 |
| *Adlercreutzia* | *Adlercreutzia_equolifaciens* | 2.84E-03 | 4.59E-04 | 2.90E-03 | 1.39E-03 | Moderate | 2.69 | 9.28E-03 | 0.01 |
| *Bacteroides* | *Bacteroides_sp._3_1_33FAA* | 9.65E-04 | 8.79E-05 | 1.70E-03 | 1.93E-04 | Moderate | 2.57 | 5.02E-05 | 0.01 |
| *Enterococcus* | *Enterococcus_faecalis* | 1.52E-03 | 1.07E-03 | 3.24E-04 | 2.08E-05 | HC | 2.88 | 1.12E-03 | 0.01 |
| *Bacteroides* | *Bacteroides_eggerthii* | 2.18E-03 | 5.78E-04 | 3.89E-03 | 1.15E-03 | Moderate | 2.89 | 3.94E-04 | 0.01 |
| *Phascolarctobacterium* | *Phascolarctobacterium_sp._CAG:207* | 3.91E-03 | 5.57E-04 | 5.52E-03 | 9.51E-04 | Moderate | 2.85 | 4.57E-03 | 0.01 |
| *Bacteroides* | *Bacteroides_vulgatus* | 2.11E-02 | 2.35E-03 | 3.91E-02 | 4.62E-03 | Moderate | 3.94 | 2.50E-05 | 0.01 |
| *Bacteroides* | *Bacteroides_caccae* | 4.45E-03 | 4.78E-04 | 6.41E-03 | 9.99E-04 | Moderate | 2.97 | 3.48E-02 | 0.01 |
| *Bacteroides* | *Bacteroides_uniformis* | 1.44E-02 | 1.50E-03 | 1.94E-02 | 2.12E-03 | Moderate | 3.29 | 3.44E-03 | 0.01 |
| *Blautia* | *Blautia_sp._Marseille-P2398* | 2.10E-03 | 2.91E-04 | 1.16E-03 | 1.41E-04 | HC | 2.66 | 1.25E-03 | 0.01 |
| *Clostridium* | *Clostridium_sp._CAG:217* | 2.58E-03 | 4.79E-04 | 1.56E-03 | 6.53E-04 | HC | 2.74 | 2.32E-02 | 0.01 |
| *Blautia* | *Blautia_sp._GD8* | 1.79E-03 | 1.71E-04 | 1.08E-03 | 1.30E-04 | HC | 2.52 | 3.69E-04 | 0.01 |
| *Bacteroides* | *Bacteroides_thetaiotaomicron* | 4.22E-03 | 4.23E-04 | 6.20E-03 | 7.72E-04 | Moderate | 2.99 | 4.95E-03 | 0.01 |
| *Bacteroides* | *Bacteroides_salyersiae* | 1.47E-03 | 2.68E-04 | 2.23E-03 | 4.34E-04 | Moderate | 2.53 | 2.07E-03 | 0.01 |
| *unclassified_p_Firmicutes* | *Firmicutes_bacterium_CAG:24* | 2.77E-03 | 3.42E-04 | 1.82E-03 | 3.66E-04 | HC | 2.75 | 1.32E-02 | 0.01 |
| *Faecalibacterium* | *Faecalibacterium_sp._CAG:82* | 2.53E-03 | 1.72E-04 | 1.79E-03 | 1.92E-04 | HC | 2.54 | 1.84E-03 | 0.01 |
| *Bacteroides* | *Bacteroides_dorei* | 6.19E-03 | 6.56E-04 | 1.06E-02 | 1.39E-03 | Moderate | 3.32 | 1.51E-04 | 0.01 |
| *Ruminococcus* | *Ruminococcus_sp._CAG:9* | 1.38E-03 | 2.09E-04 | 7.14E-04 | 9.52E-05 | HC | 2.51 | 1.26E-03 | 0.01 |
| *Anaerostipes* | *Anaerostipes_hadrus* | 5.24E-03 | 6.57E-04 | 4.24E-03 | 1.04E-03 | HC | 2.80 | 4.29E-03 | 0.01 |
| *Ruminococcus* | *Ruminococcus_sp._5_1_39BFAA* | 2.27E-03 | 3.20E-04 | 1.25E-03 | 1.51E-04 | HC | 2.70 | 2.18E-03 | 0.01 |
| *Bacteroides* | *Bacteroides_xylanisolvens* | 2.02E-03 | 2.16E-04 | 2.84E-03 | 4.80E-04 | Moderate | 2.63 | 2.34E-02 | 0.01 |
| *Blautia* | *Blautia_obeum* | 1.10E-02 | 8.89E-04 | 6.57E-03 | 5.83E-04 | HC | 3.33 | 1.65E-04 | 0.01 |
| *Dorea* | *Dorea_longicatena* | 1.20E-02 | 1.12E-03 | 1.13E-02 | 3.08E-03 | HC | 3.11 | 1.03E-02 | 0.01 |
| *Ruminococcus* | *Ruminococcus_sp._CAG:17* | 1.44E-03 | 2.78E-04 | 5.79E-04 | 7.62E-05 | HC | 2.61 | 1.99E-04 | 0.01 |
| *Clostridium* | *Clostridium_sp._CAG:510* | 7.51E-04 | 4.77E-04 | 2.01E-04 | 2.37E-05 | HC | 2.55 | 1.86E-02 | 0.01 |
| *Bacteroides* | *Bacteroides_fragilis* | 6.30E-03 | 4.51E-04 | 9.76E-03 | 9.50E-04 | Moderate | 3.22 | 2.17E-04 | 0.01 |
| *Eubacterium* | *Eubacterium_sp._CAG:115* | 1.16E-03 | 3.60E-04 | 3.24E-04 | 1.37E-04 | HC | 2.66 | 3.40E-02 | 0.01 |
| *Lactobacillus* | *Lactobacillus_ruminis* | 1.54E-03 | 6.94E-04 | 1.59E-04 | 3.01E-05 | HC | 2.83 | 1.11E-02 | 0.01 |
| *Faecalibacterium* | *Faecalibacterium_sp._CAG:74* | 4.94E-03 | 1.11E-03 | 1.48E-03 | 3.91E-04 | HC | 3.32 | 1.50E-02 | 0.01 |
| *Subdoligranulum* | *Subdoligranulum_variabile* | 2.27E-02 | 1.91E-03 | 1.32E-02 | 1.74E-03 | HC | 3.68 | 2.36E-03 | 0.01 |
| *Blautia* | *Blautia_wexlerae* | 9.09E-03 | 1.18E-03 | 5.36E-03 | 5.93E-04 | HC | 3.26 | 1.56E-03 | 0.01 |
| *Bacteroides* | *Bacteroides_ovatus* | 6.49E-03 | 7.48E-04 | 8.33E-03 | 9.67E-04 | Moderate | 2.97 | 5.19E-03 | 0.01 |
| *Blautia* | *Blautia_sp._CAG:237* | 2.27E-03 | 9.41E-04 | 8.18E-04 | 1.50E-04 | HC | 2.83 | 9.34E-03 | 0.01 |
| *Faecalibacterium* | *Faecalibacterium_prausnitzii* | 5.83E-02 | 4.20E-03 | 4.20E-02 | 4.13E-03 | HC | 3.89 | 1.08E-02 | 0.01 |

**Supplementary Table 4. Discriminatory bacteria species between HCs and severe group.**

| Genus | Species | Relative abundance | | | | Enrichment | LDA | *p* value | Importance |
| --- | --- | --- | --- | --- | --- | --- | --- | --- | --- |
|  |  | HCs | | Severe | |  |  |  |  |
|  |  | mean | sem | mean | sem |  |  |  |  |
| *Bacteroides* | *Bacteroides_faecis* | 8.02E-04 | 1.36E-04 | 1.89E-03 | 3.90E-04 | Severe | 2.72 | 2.96E-04 | 0.04 |
| *Eubacterium* | *Eubacterium_hallii* | 9.44E-03 | 1.06E-03 | 3.61E-03 | 7.28E-04 | HC | 3.47 | 8.08E-05 | 0.04 |
| *unclassified_p_Firmicutes* | *Firmicutes_bacterium_CAG:41* | 5.95E-03 | 7.58E-04 | 2.42E-03 | 4.32E-04 | HC | 3.24 | 1.73E-04 | 0.04 |
| *Clostridium* | *Clostridium_sp._CAG:417* | 1.19E-03 | 4.34E-04 | 9.49E-04 | 5.70E-04 | HC | 2.62 | 1.13E-03 | 0.03 |
| *Eubacterium* | *Eubacterium_hallii_CAG:12* | 1.64E-03 | 1.94E-04 | 5.76E-04 | 1.32E-04 | HC | 2.73 | 7.48E-05 | 0.03 |
| *Ruminococcus* | *Ruminococcus_sp._CAG:9* | 1.38E-03 | 2.09E-04 | 6.12E-04 | 1.17E-04 | HC | 2.58 | 3.00E-04 | 0.03 |
| *Dorea* | *Dorea_longicatena* | 1.20E-02 | 1.12E-03 | 8.23E-03 | 1.44E-03 | HC | 3.31 | 6.99E-03 | 0.03 |
| *Ruminococcus* | *Ruminococcus_gnavus_CAG:126* | 2.64E-04 | 5.75E-05 | 8.90E-04 | 5.36E-04 | Severe | 2.50 | 1.07E-03 | 0.02 |
| *Bifidobacterium* | *Bifidobacterium_longum* | 5.61E-03 | 6.72E-04 | 1.20E-02 | 2.61E-03 | Severe | 3.44 | 1.79E-02 | 0.02 |
| *Blautia* | *Blautia_sp._Marseille-P2398* | 2.10E-03 | 2.91E-04 | 9.89E-04 | 1.77E-04 | HC | 2.74 | 1.93E-04 | 0.02 |
| *Bacteroides* | *Bacteroides_dorei* | 6.19E-03 | 6.56E-04 | 1.28E-02 | 2.23E-03 | Severe | 3.53 | 5.28E-04 | 0.02 |
| *Blautia* | *Blautia_obeum* | 1.10E-02 | 8.89E-04 | 8.73E-03 | 2.29E-03 | HC | 3.19 | 1.54E-03 | 0.02 |
| *Ruminococcus* | *Ruminococcus_sp._5_1_39BFAA* | 2.27E-03 | 3.20E-04 | 1.09E-03 | 1.97E-04 | HC | 2.76 | 2.79E-04 | 0.02 |
| *Bifidobacterium* | *Bifidobacterium_breve* | 4.30E-04 | 4.51E-05 | 1.27E-03 | 5.04E-04 | Severe | 2.64 | 2.41E-02 | 0.02 |
| *Blautia* | *Blautia_sp._CAG:37* | 3.68E-03 | 3.38E-04 | 2.72E-03 | 5.53E-04 | HC | 2.77 | 3.89E-02 | 0.02 |
| *Bacteroides* | *Bacteroides_sp._3_1_40A* | 7.30E-04 | 7.32E-05 | 1.36E-03 | 2.08E-04 | Severe | 2.52 | 5.28E-04 | 0.02 |
| *Dorea* | *Dorea_sp._CAG:105* | 1.76E-03 | 3.39E-04 | 1.72E-03 | 6.62E-04 | HC | 2.60 | 5.76E-03 | 0.02 |
| *Eubacterium* | *Eubacterium_sp._CAG:202* | 7.15E-03 | 1.91E-03 | 8.41E-04 | 5.77E-04 | HC | 3.46 | 1.35E-04 | 0.02 |
| *Bacteroides* | *Bacteroides_vulgatus* | 2.11E-02 | 2.35E-03 | 4.29E-02 | 7.34E-03 | Severe | 4.04 | 3.33E-04 | 0.02 |
| *Bacteroides* | *Bacteroides_sp._CAG:98* | 8.64E-04 | 2.16E-04 | 2.35E-03 | 7.16E-04 | Severe | 2.85 | 7.25E-03 | 0.02 |
| *Blautia* | *Blautia_wexlerae* | 9.09E-03 | 1.18E-03 | 4.65E-03 | 7.09E-04 | HC | 3.34 | 4.35E-04 | 0.02 |
| *Fusicatenibacter* | *Fusicatenibacter_saccharivorans* | 8.34E-03 | 6.38E-04 | 6.29E-03 | 1.10E-03 | HC | 3.09 | 1.58E-02 | 0.02 |
| *Eubacterium* | *Eubacterium_sp._CAG:146* | 1.92E-03 | 2.58E-04 | 2.34E-03 | 7.63E-04 | Severe | 2.51 | 1.82E-02 | 0.02 |
| *Sutterella* | *Sutterella_wadsworthensis* | 6.20E-04 | 1.55E-04 | 1.75E-03 | 5.87E-04 | Severe | 2.73 | 2.55E-03 | 0.02 |
| *Bacteroides* | *Bacteroides_sp._3_1_33FAA* | 9.65E-04 | 8.79E-05 | 1.88E-03 | 2.75E-04 | Severe | 2.68 | 2.25E-04 | 0.02 |
| *Bacteroides* | *Bacteroides_sp._4_3_47FAA* | 7.96E-04 | 8.18E-05 | 1.63E-03 | 2.94E-04 | Severe | 2.61 | 3.61E-04 | 0.01 |
| *Blautia* | *Ruminococcus_gnavus* | 3.84E-03 | 6.11E-04 | 8.86E-03 | 4.18E-03 | Severe | 3.40 | 2.70E-02 | 0.01 |
| *Eubacterium* | *Eubacterium_sp._CAG:156* | 1.42E-03 | 3.26E-04 | 3.20E-04 | 9.80E-05 | HC | 2.74 | 1.10E-03 | 0.01 |
| *Veillonella* | *Veillonella_sp._CAG:933* | 1.32E-03 | 3.80E-04 | 2.35E-04 | 4.82E-05 | HC | 2.80 | 2.19E-03 | 0.01 |
| *Prevotella* | *Prevotella_copri_CAG:164* | 8.54E-03 | 1.78E-03 | 1.83E-02 | 6.17E-03 | Severe | 3.69 | 2.63E-02 | 0.01 |
| *Clostridium* | *Clostridium_sp._CAG:62* | 1.16E-03 | 2.73E-04 | 3.73E-04 | 1.64E-04 | HC | 2.66 | 1.56E-03 | 0.01 |
| *Bacteroides* | *Bacteroides_massiliensis* | 3.03E-03 | 5.36E-04 | 7.28E-03 | 1.80E-03 | Severe | 3.31 | 6.92E-04 | 0.01 |
| *unclassified_f_Lachnospiraceae* | *Eubacterium_rectale* | 2.89E-02 | 3.03E-03 | 1.99E-02 | 4.21E-03 | HC | 3.68 | 2.74E-02 | 0.01 |
| *Eubacterium* | *Eubacterium_ventriosum* | 3.35E-03 | 3.31E-04 | 1.70E-03 | 2.17E-04 | HC | 2.93 | 1.11E-03 | 0.01 |
| *Ruminococcus* | *Ruminococcus_callidus* | 2.77E-03 | 4.19E-04 | 9.70E-04 | 2.77E-04 | HC | 2.93 | 1.03E-03 | 0.01 |
| *Ruminococcus* | *Ruminococcus_sp._JC304* | 1.42E-03 | 1.26E-04 | 7.75E-04 | 1.03E-04 | HC | 2.52 | 3.79E-04 | 0.01 |
| *Klebsiella* | *Klebsiella_pneumoniae* | 7.10E-03 | 2.23E-03 | 1.59E-03 | 8.23E-04 | HC | 3.50 | 2.99E-03 | 0.01 |
| *Enterobacter* | *Enterobacter_cloacae* | 1.65E-03 | 6.76E-04 | 2.84E-04 | 9.39E-05 | HC | 2.91 | 3.47E-03 | 0.01 |
| *Bacteroides* | *Bacteroides_sp._D2* | 5.20E-04 | 5.68E-05 | 1.17E-03 | 3.73E-04 | Severe | 2.55 | 2.26E-03 | 0.01 |
| *Bacteroides* | *Bacteroides_sp._9_1_42FAA* | 6.62E-04 | 6.11E-05 | 1.30E-03 | 1.94E-04 | Severe | 2.52 | 4.82E-04 | 0.01 |
| *Citrobacter* | *Citrobacter_freundii* | 9.81E-04 | 6.09E-04 | 1.56E-04 | 6.82E-05 | HC | 2.73 | 1.78E-02 | 0.01 |
| *Dorea* | *Dorea_formicigenerans* | 2.70E-03 | 1.63E-04 | 1.90E-03 | 1.85E-04 | HC | 2.64 | 1.10E-02 | 0.01 |
| *Coprococcus* | *Coprococcus_sp._ART55/1* | 1.41E-03 | 2.96E-04 | 5.30E-04 | 1.61E-04 | HC | 2.65 | 5.98E-03 | 0.01 |
| *Bacteroides* | *Bacteroides_ovatus* | 6.49E-03 | 7.48E-04 | 1.16E-02 | 2.19E-03 | Severe | 3.41 | 2.10E-03 | 0.01 |
| *Eubacterium* | *Eubacterium_rectale_CAG:36* | 2.44E-03 | 2.74E-04 | 1.62E-03 | 3.45E-04 | HC | 2.64 | 4.50E-02 | 0.01 |
| *Subdoligranulum* | *Subdoligranulum_variabile* | 2.27E-02 | 1.91E-03 | 1.48E-02 | 3.19E-03 | HC | 3.65 | 6.32E-03 | 0.01 |
| *Eubacterium* | *Eubacterium_sp._CAG:180* | 7.71E-03 | 1.63E-03 | 6.64E-03 | 4.07E-03 | HC | 3.29 | 2.99E-03 | 0.01 |
| *Bacteroides* | *Bacteroides_xylanisolvens* | 2.02E-03 | 2.16E-04 | 3.58E-03 | 7.96E-04 | Severe | 2.88 | 2.18E-02 | 0.01 |
| *Ruminococcus* | *Ruminococcus_bromii* | 8.07E-03 | 1.21E-03 | 3.18E-03 | 1.07E-03 | HC | 3.38 | 2.99E-03 | 0.01 |
| *Ruminococcus* | *Ruminococcus_sp._CAG:108* | 8.53E-03 | 1.30E-03 | 3.11E-03 | 1.01E-03 | HC | 3.42 | 1.62E-03 | 0.01 |
| *Roseburia* | *Roseburia_intestinalis* | 5.87E-03 | 6.43E-04 | 4.31E-03 | 1.05E-03 | HC | 2.97 | 5.76E-03 | 0.01 |
| *Bacteroides* | *Bacteroides_stercoris* | 5.81E-03 | 1.04E-03 | 7.96E-03 | 2.04E-03 | Severe | 3.10 | 4.28E-02 | 0.01 |
| *Parabacteroides* | *Parabacteroides_distasonis* | 2.57E-03 | 2.25E-04 | 3.77E-03 | 5.78E-04 | Severe | 2.76 | 2.63E-03 | 0.01 |
| *Anaerostipes* | *Anaerostipes_hadrus* | 5.24E-03 | 6.57E-04 | 2.86E-03 | 4.89E-04 | HC | 3.06 | 3.32E-02 | 0.01 |
| *unclassified_p_Firmicutes* | *Firmicutes_bacterium_CAG:227* | 1.35E-03 | 2.58E-04 | 7.69E-04 | 2.90E-04 | HC | 2.55 | 1.06E-02 | 0.01 |
| *Bacteroides* | *Bacteroides_thetaiotaomicron* | 4.22E-03 | 4.23E-04 | 7.69E-03 | 1.61E-03 | Severe | 3.23 | 7.73E-03 | 0.01 |
| *Bacteroides* | *Bacteroides_fragilis* | 6.30E-03 | 4.51E-04 | 9.44E-03 | 1.37E-03 | Severe | 3.23 | 1.31E-02 | 0.01 |
| *Bacteroides* | *Bacteroides_coprophilus* | 1.69E-03 | 3.43E-04 | 2.22E-03 | 7.68E-04 | Severe | 2.60 | 2.72E-02 | 0.01 |
| *unclassified_p_Firmicutes* | *Firmicutes_bacterium_CAG:56* | 2.70E-03 | 6.33E-04 | 1.40E-03 | 3.66E-04 | HC | 2.75 | 4.37E-02 | 0.01 |
| *Blautia* | *Blautia_sp._CAG:237* | 2.27E-03 | 9.41E-04 | 1.23E-03 | 3.95E-04 | HC | 2.70 | 4.91E-02 | 0.01 |
| *Ruminococcus* | *Ruminococcus_sp._CAG:177* | 2.00E-03 | 3.45E-04 | 1.18E-03 | 4.50E-04 | HC | 2.68 | 3.17E-02 | 0.01 |
| *Clostridium* | *Clostridium_sp._L2-50* | 1.33E-03 | 2.00E-04 | 5.29E-04 | 4.12E-05 | HC | 2.66 | 7.81E-04 | 0.01 |
| *Clostridium* | *Clostridium_sp._CAG:510* | 7.51E-04 | 4.77E-04 | 1.54E-04 | 1.69E-05 | HC | 2.62 | 3.47E-03 | 0.01 |
| *Eubacterium* | *Eubacterium_sp._CAG:115* | 1.16E-03 | 3.60E-04 | 5.14E-05 | 8.19E-06 | HC | 2.77 | 1.67E-03 | 0.01 |
| *Bacteroides* | *Bacteroides_sp._3_1_13* | 1.02E-03 | 1.18E-04 | 1.73E-03 | 3.49E-04 | Severe | 2.54 | 8.38E-03 | 0.01 |
| *Clostridium* | *Clostridium_sp._CAG:264* | 8.16E-04 | 1.53E-04 | 1.21E-04 | 2.86E-05 | HC | 2.58 | 1.10E-02 | 0.01 |
| *Clostridium* | *Clostridium_sp._CAG:75* | 1.39E-03 | 5.70E-04 | 4.77E-04 | 1.81E-04 | HC | 2.71 | 4.70E-02 | 0.01 |
| *Clostridium* | *Clostridium_sp._CAG:217* | 2.58E-03 | 4.79E-04 | 1.10E-03 | 3.66E-04 | HC | 2.84 | 2.85E-02 | <0.01 |
| *Coprococcus* | *Coprococcus_eutactus* | 3.62E-03 | 6.78E-04 | 1.42E-03 | 3.16E-04 | HC | 3.05 | 1.14E-02 | <0.02 |
| *Faecalibacterium* | *Faecalibacterium_sp._CAG:82* | 2.53E-03 | 1.72E-04 | 1.87E-03 | 2.81E-04 | HC | 2.54 | 2.49E-02 | <0.03 |
| *Ruminiclostridium* | *Eubacterium_siraeum* | 1.17E-03 | 1.72E-04 | 5.30E-04 | 7.28E-05 | HC | 2.55 | 5.70E-03 | <0.04 |
| *Oscillibacter* | *Oscillibacter_sp._ER4* | 4.09E-03 | 3.60E-04 | 2.70E-03 | 6.88E-04 | HC | 2.83 | 1.38E-02 | <0.05 |
| *Bacteroides* | *Bacteroides_eggerthii* | 2.18E-03 | 5.78E-04 | 3.59E-03 | 1.28E-03 | Severe | 2.99 | 4.80E-02 | <0.06 |
| *Faecalibacterium* | *Faecalibacterium_prausnitzii* | 5.83E-02 | 4.20E-03 | 4.00E-02 | 4.67E-03 | HC | 3.97 | 4.84E-02 | <0.07 |

**Supplementary Table 5. Discriminatory KOs between HCs and moderate group.**

| KO | Description | Relative abundance | | | | Enrichment | LDA | *p* |
| --- | --- | --- | --- | --- | --- | --- | --- | --- |
|  |  | HCs | | Moderate | |  |  |  |
|  |  | mean | sem | mean | sem |  |  |  |
| K03088 | RNA polymerase sigma-70 factor, ECF subfamily | 5.25E-03 | 1.60E-04 | 6.37E-03 | 3.17E-04 | Moderate | 2.90 | 3.75E-04 |
| K21572 | Starch-binding outer membrane protein, SusD/RagB family | 1.17E-02 | 8.57E-04 | 1.95E-02 | 1.80E-03 | Moderate | 3.30 | 7.67E-05 |
| K07133 | Uncharacterized protein | 3.63E-03 | 1.87E-04 | 4.81E-03 | 5.49E-04 | Moderate | 2.58 | 4.01E-02 |

**Supplementary Table 6. Discriminatory KOs between HCs and severe group.**

| KO | Description | Relative abundance | | | | Enrichment | LDA | *p* |
| --- | --- | --- | --- | --- | --- | --- | --- | --- |
|  |  | HCs | | Severe | |  |  |  |
|  |  | mean | sem | mean | sem |  |  |  |
| K03088 | RNA polymerase sigma-70 factor, ECF subfamily | 5.25E-03 | 1.60E-04 | 6.37E-03 | 3.17E-04 | Severe | 2.91 | 1.59E-03 |
| K01190 | Beta-galactosidase | 5.53E-03 | 1.73E-04 | 6.90E-03 | 3.24E-04 | Severe | 2.54 | 9.31E-04 |
| K21572 | Starch-binding outer membrane protein, SusD/RagB family | 1.17E-02 | 8.57E-04 | 1.95E-02 | 1.80E-03 | Severe | 3.37 | 7.97E-05 |
| K12373 | Hexosaminidase | 2.01E-03 | 1.30E-04 | 3.07E-03 | 2.66E-04 | Severe | 2.53 | 1.25E-04 |
| K07133 | Uncharacterized protein | 3.63E-03 | 1.87E-04 | 4.81E-03 | 5.49E-04 | Severe | 2.67 | 2.07E-02 |

**Supplementary Table 7. Potential biomarker for identifying different severity of MDD**

| Family | Genus | Species | Enrichment | Gini importance | | |
| --- | --- | --- | --- | --- | --- | --- |
|  |  |  |  | HCs vs Mild | HCs vs Moderate | HCs vs Severe |
| *Eubacteriaceae* | *Eubacterium* | *[Eubacterium]_hallii* | HC | 0.071408 | 0.020529 | 0.038106 |
| *Bacteroidaceae* | *Bacteroides* | *Bacteroides_coprophilus* | Mild | 0.094479 | NA | NA |
| *Bacteroidaceae* | *Bacteroides* | *Bacteroides_dorei* | Severe | NA | NA | 0.020943 |
| *Bacteroidaceae* | *Bacteroides* | *Bacteroides_faecis* | Severe | NA | NA | 0.04472 |
| *Bacteroidaceae* | *Bacteroides* | *Bacteroides_massiliensis* | Moderate | NA | 0.023398 | NA |
| *Bacteroidaceae* | *Bacteroides* | *Bacteroides_sp._CAG:98* | Moderate | NA | 0.02644 | NA |
| *Bacteroidaceae* | *Bacteroides* | *Bacteroides_stercoris* | Moderate | NA | 0.037646 | NA |
| *Bacteroidaceae* | *Bacteroides* | *Bacteroides_stercoris_CAG:120* | Moderate | NA | 0.032498 | NA |
| *Bifidobacteriaceae* | *Bifidobacterium* | *Bifidobacterium_longum* | Mild&Severe | 0.082395 | NA | 0.023765 |
| *Desulfovibrionaceae* | *Bilophila* | *Bilophila_wadsworthia* | Moderate | NA | 0.027305 | NA |
| *Lachnospiraceae* | *Blautia* | *Blautia_obeum* | HC | NA | NA | 0.020914 |
| *Lachnospiraceae* | *Blautia* | *Blautia_sp._CAG:237* | HC | 0.058668 | NA | NA |
| *Lachnospiraceae* | *Blautia* | *Blautia_sp._GD8* | HC | 0.052819 | NA | NA |
| *Lachnospiraceae* | *Blautia* | *Blautia_sp._KLE_1732* | HC | 0.054307 | NA | NA |
| *Lachnospiraceae* | *Blautia* | *Blautia_sp._Marseille-P2398* | HC | NA | NA | 0.023021 |
| *unclassified_o__Clostridiales* | *unclassified_o__Clostridiales* | *butyrate-producing_bacterium_SS3/4* | Moderate | NA | 0.024217 | NA |
| *Clostridiaceae* | *Clostridium* | *Clostridium_sp._CAG:217* | HC | 0.040493 | NA | NA |
| *Clostridiaceae* | *Clostridium* | *Clostridium_sp._CAG:302* | HC | 0.086884 | 0.023607 | NA |
| *Clostridiaceae* | *Clostridium* | *Clostridium_sp._CAG:417* | HC | NA | NA | 0.030688 |
| *Clostridiaceae* | *Clostridium* | *Clostridium_sp._CAG:75* | HC | NA | 0.021457 | NA |
| *Lachnospiraceae* | *Dorea* | *Dorea_longicatena* | HC | NA | NA | 0.025027 |
| *Lachnospiraceae* | *Dorea* | *Dorea_sp._CAG:105* | HC | 0.065004 | NA | NA |
| *Enterobacteriaceae* | *Enterobacter* | *Enterobacter_cloacae* | HC | NA | 0.044883 | NA |
| *Enterobacteriaceae* | *Enterobacter* | *Enterobacter_sp._GN02315* | HC | NA | 0.023771 | NA |
| *Eubacteriaceae* | *Eubacterium* | *Eubacterium_hallii_CAG:12* | HC | 0.067791 | NA | 0.030361 |
| *Eubacteriaceae* | *Eubacterium* | *Eubacterium_sp._CAG:146* | Moderate | NA | 0.03244 | NA |
| *Eubacteriaceae* | *Eubacterium* | *Eubacterium_sp._CAG:156* | HC | 0.081436 | NA | NA |
| *unclassified_p__Firmicutes* | *unclassified_p__Firmicutes* | *Firmicutes_bacterium_CAG:110* | HC | 0.049392 | NA | NA |
| *unclassified_p__Firmicutes* | *unclassified_p__Firmicutes* | *Firmicutes_bacterium_CAG:227* | HC | NA | 0.022149 | NA |
| *unclassified_p__Firmicutes* | *unclassified_p__Firmicutes* | *Firmicutes_bacterium_CAG:41* | HC | NA | 0.028162 | 0.035282 |
| *Enterobacteriaceae* | *Klebsiella* | *Klebsiella_pneumoniae* | HC | NA | 0.067364 | NA |
| *Sutterellaceae* | *Parasutterella* | *Parasutterella_excrementihominis* | Mild | 0.106408 | NA | NA |
| *Ruminococcaceae* | *Ruminococcus* | *Ruminococcus_gnavus_CAG:126* | Severe | NA | NA | 0.024862 |
| *Ruminococcaceae* | *Ruminococcus* | *Ruminococcus_sp._5_1_39BFAA* | HC | NA | NA | 0.020388 |
| *Ruminococcaceae* | *Ruminococcus* | *Ruminococcus_sp._CAG:17* | HC | 0.088515 | NA | NA |
| *Ruminococcaceae* | *Ruminococcus* | *Ruminococcus_sp._CAG:9* | HC | NA | NA | 0.025829 |
| *Enterobacteriaceae* | *Shigella* | *Shigella_sonnei* | HC | NA | 0.032997 | NA |

**Supplementary Table 8. Discriminatory bacteria between HCs and MDD in female samples**

| Genus | Species | Relative abundance | | | | Enrichment | LDA | *p* value |
| --- | --- | --- | --- | --- | --- | --- | --- | --- |
|  |  | HCs | | MDD | |  |  |  |
|  |  | mean | sem | mean | sem |  |  |  |
| Clostridium | Clostridium_sp._CAG:217 | 2.81E-03 | 6.79E-04 | 9.53E-04 | 2.22E-04 | HCs | 2.98 | 8.18E-03 |
| Roseburia | Roseburia_intestinalis | 6.43E-03 | 9.82E-04 | 4.27E-03 | 4.89E-04 | HCs | 3.02 | 3.83E-02 |
| Butyricimonas | Butyricimonas_virosa | 1.12E-03 | 1.45E-04 | 1.77E-03 | 2.21E-04 | MDD | 2.51 | 2.19E-02 |
| Faecalibacterium | Faecalibacterium_prausnitzii | 6.01E-02 | 6.34E-03 | 3.84E-02 | 2.98E-03 | HCs | 4.09 | 2.49E-02 |
| Clostridium | Clostridium_sp._CAG:510 | 1.14E-03 | 8.11E-04 | 2.07E-04 | 2.05E-05 | HCs | 2.71 | 3.60E-03 |
| Bacteroides | Bacteroides_vulgatus | 2.72E-02 | 3.52E-03 | 4.03E-02 | 4.29E-03 | MDD | 3.82 | 4.97E-03 |
| Bacteroides | Bacteroides_salyersiae | 1.45E-03 | 2.70E-04 | 2.32E-03 | 3.83E-04 | MDD | 2.68 | 1.46E-03 |
| Faecalibacterium | Faecalibacterium_sp._CAG:82 | 2.65E-03 | 2.55E-04 | 1.85E-03 | 1.84E-04 | HCs | 2.67 | 8.11E-03 |
| Bacteroides | Bacteroides_stercoris | 7.79E-03 | 1.62E-03 | 1.65E-02 | 2.48E-03 | MDD | 3.66 | 1.90E-03 |
| Bacteroides | Bacteroides_thetaiotaomicron | 5.08E-03 | 5.42E-04 | 7.07E-03 | 8.08E-04 | MDD | 3.03 | 1.69E-02 |
| Bacteroides | Bacteroides_massiliensis | 3.62E-03 | 8.36E-04 | 8.32E-03 | 1.42E-03 | MDD | 3.40 | 1.85E-03 |
| Eubacterium | Eubacterium_ventriosum | 3.40E-03 | 4.09E-04 | 2.31E-03 | 4.22E-04 | HCs | 2.88 | 1.93E-04 |
| Bacteroides | Bacteroides_stercoris_CAG:120 | 1.51E-03 | 3.35E-04 | 3.22E-03 | 5.20E-04 | MDD | 2.95 | 9.07E-03 |
| Ruminiclostridium | Eubacterium_siraeum | 1.52E-03 | 2.84E-04 | 2.41E-03 | 7.96E-04 | MDD | 2.68 | 2.77E-02 |
| Blautia | Blautia_obeum | 9.26E-03 | 9.13E-04 | 7.30E-03 | 1.16E-03 | HCs | 3.06 | 6.57E-03 |
| Parabacteroides | Parabacteroides_distasonis | 3.35E-03 | 3.30E-04 | 4.19E-03 | 3.99E-04 | MDD | 2.66 | 3.19E-02 |
| Eubacterium | Eubacterium_hallii | 9.04E-03 | 1.40E-03 | 3.42E-03 | 5.42E-04 | HCs | 3.51 | 3.37E-05 |
| Bacteroides | Bacteroides_dorei | 7.98E-03 | 9.36E-04 | 1.19E-02 | 1.37E-03 | MDD | 3.34 | 1.08E-02 |
| Clostridium | Clostridium_sp._CAG:7 | 3.16E-03 | 5.15E-04 | 6.09E-03 | 1.21E-03 | MDD | 3.15 | 4.29E-02 |
| Bacteroides | Bacteroides_fragilis | 7.36E-03 | 5.80E-04 | 1.05E-02 | 9.58E-04 | MDD | 3.22 | 1.30E-02 |
| Dorea | Dorea_formicigenerans | 2.53E-03 | 2.04E-04 | 1.93E-03 | 1.31E-04 | HCs | 2.53 | 2.14E-02 |
| Blautia | Blautia_wexlerae | 7.48E-03 | 1.06E-03 | 4.92E-03 | 5.38E-04 | HCs | 3.20 | 4.10E-03 |
| Anaerostipes | Anaerostipes_hadrus | 5.41E-03 | 9.81E-04 | 3.47E-03 | 5.47E-04 | HCs | 3.05 | 2.38E-02 |
| Bacteroides | Bacteroides_sp._3_1_33FAA | 1.19E-03 | 1.25E-04 | 1.84E-03 | 1.85E-04 | MDD | 2.57 | 3.91E-03 |
| Eubacterium | Eubacterium_hallii_CAG:12 | 1.58E-03 | 2.58E-04 | 5.63E-04 | 1.00E-04 | HCs | 2.78 | 5.38E-05 |
| Bacteroides | Bacteroides_sp._CAG:98 | 1.08E-03 | 3.40E-04 | 2.86E-03 | 5.84E-04 | MDD | 2.98 | 3.56E-03 |
| Blautia | Blautia_sp._Marseille-P2398 | 1.63E-03 | 2.50E-04 | 1.03E-03 | 1.29E-04 | HCs | 2.57 | 3.09E-03 |
| Bacteroides | Bacteroides_ovatus | 7.91E-03 | 1.01E-03 | 1.04E-02 | 1.25E-03 | MDD | 3.14 | 3.12E-02 |
| Eubacterium | Eubacterium_sp._CAG:202 | 4.47E-03 | 1.77E-03 | 3.39E-03 | 1.03E-03 | HCs | 2.96 | 3.59E-02 |
| Akkermansia | Akkermansia_muciniphila_CAG:154 | 1.16E-03 | 3.83E-04 | 6.10E-04 | 2.97E-04 | HCs | 2.55 | 4.69E-02 |
| Ruminococcus | Ruminococcus_sp._5_1_39BFAA | 1.77E-03 | 2.80E-04 | 1.15E-03 | 1.44E-04 | HCs | 2.59 | 7.37E-03 |
| Eubacterium | Eubacterium_sp._CAG:156 | 1.90E-03 | 5.33E-04 | 1.64E-03 | 6.42E-04 | HCs | 2.64 | 1.53E-02 |
| Clostridium | Clostridium_sp._CAG:417 | 1.55E-03 | 6.72E-04 | 1.12E-03 | 5.58E-04 | HCs | 2.56 | 2.46E-02 |
| Dorea | Dorea_longicatena | 1.03E-02 | 1.31E-03 | 7.60E-03 | 7.71E-04 | HCs | 3.17 | 1.08E-02 |
| Subdoligranulum | Subdoligranulum_variabile | 2.13E-02 | 2.47E-03 | 1.45E-02 | 1.85E-03 | HCs | 3.58 | 2.75E-02 |
| Klebsiella | Klebsiella_pneumoniae | 2.39E-03 | 5.71E-04 | 1.13E-03 | 3.67E-04 | HCs | 2.74 | 8.09E-05 |
| Eubacterium | Eubacterium_sp._CAG:115 | 1.19E-03 | 4.04E-04 | 4.47E-04 | 1.58E-04 | HCs | 2.66 | 6.63E-03 |
| unclassified_p__Firmicutes | Firmicutes_bacterium_CAG:41 | 6.96E-03 | 1.20E-03 | 4.28E-03 | 7.72E-04 | HCs | 3.12 | 4.89E-04 |

**Supplementary Table 9. Discriminatory bacteria between HCs and MDD in male samples**

| Genus | Species | Relative abundance | | | | Enrichment | LDA | *p* value |
| --- | --- | --- | --- | --- | --- | --- | --- | --- |
|  |  | HCs | | MDD | |  |  |  |
|  |  | mean | sem | mean | sem |  |  |  |
| Eubacterium | Eubacterium_hallii | 1.00E-02 | 1.61E-03 | 4.65E-03 | 8.98E-04 | HCs | 3.50 | 1.47E-03 |
| Blautia | Ruminococcus_gnavus | 3.81E-03 | 1.06E-03 | 9.26E-03 | 3.50E-03 | MDD | 3.42 | 2.56E-02 |
| Adlercreutzia | Adlercreutzia_equolifaciens | 3.96E-03 | 9.55E-04 | 2.06E-03 | 6.56E-04 | HCs | 3.00 | 1.07E-02 |
| Anaerostipes | Anaerostipes_hadrus | 5.00E-03 | 7.73E-04 | 3.88E-03 | 1.25E-03 | HCs | 2.88 | 1.50E-02 |
| Bacteroides | Bacteroides_caccae | 2.42E-03 | 6.07E-04 | 3.83E-03 | 6.76E-04 | MDD | 2.83 | 1.10E-02 |
| Bacteroides | Bacteroides_dorei | 3.64E-03 | 7.72E-04 | 9.34E-03 | 1.64E-03 | MDD | 3.46 | 3.73E-04 |
| Bacteroides | Bacteroides_eggerthii | 2.19E-03 | 1.23E-03 | 3.61E-03 | 1.08E-03 | MDD | 2.91 | 2.39E-03 |
| Bacteroides | Bacteroides_finegoldii | 1.45E-03 | 3.49E-04 | 2.23E-03 | 4.17E-04 | MDD | 2.68 | 5.60E-03 |
| Bacteroides | Bacteroides_fragilis | 4.79E-03 | 6.78E-04 | 8.02E-03 | 1.31E-03 | MDD | 3.24 | 5.60E-03 |
| Bacteroides | Bacteroides_massiliensis | 2.19E-03 | 5.12E-04 | 8.85E-03 | 2.68E-03 | MDD | 3.53 | 1.95E-03 |
| Bacteroides | Bacteroides_ovatus | 4.47E-03 | 1.05E-03 | 6.95E-03 | 1.03E-03 | MDD | 3.08 | 6.00E-03 |
| Bacteroides | Bacteroides_sp._3_1_33FAA | 6.39E-04 | 1.07E-04 | 1.45E-03 | 2.07E-04 | MDD | 2.63 | 1.67E-04 |
| Bacteroides | Bacteroides_sp._3_1_40A | 4.66E-04 | 7.88E-05 | 1.24E-03 | 1.93E-04 | MDD | 2.60 | 6.02E-04 |
| Bacteroides | Bacteroides_sp._4_3_47FAA | 5.45E-04 | 9.76E-05 | 1.44E-03 | 2.45E-04 | MDD | 2.65 | 5.00E-04 |
| Bacteroides | Bacteroides_sp._9_1_42FAA | 4.05E-04 | 6.38E-05 | 1.03E-03 | 1.58E-04 | MDD | 2.53 | 5.90E-04 |
| Bacteroides | Bacteroides_sp._CAG:98 | 5.55E-04 | 1.97E-04 | 3.05E-03 | 1.12E-03 | MDD | 3.09 | 3.30E-02 |
| Bacteroides | Bacteroides_stercoris | 3.01E-03 | 9.35E-04 | 1.18E-02 | 3.23E-03 | MDD | 3.68 | 2.56E-02 |
| Bacteroides | Bacteroides_thetaiotaomicron | 3.01E-03 | 6.51E-04 | 5.61E-03 | 1.15E-03 | MDD | 3.08 | 1.55E-02 |
| Bacteroides | Bacteroides_uniformis | 9.47E-03 | 2.35E-03 | 1.17E-02 | 1.76E-03 | MDD | 3.19 | 9.22E-03 |
| Bacteroides | Bacteroides_vulgatus | 1.24E-02 | 2.33E-03 | 3.76E-02 | 6.33E-03 | MDD | 4.11 | 2.33E-04 |
| Bacteroides | Bacteroides_xylanisolvens | 1.31E-03 | 2.92E-04 | 2.03E-03 | 2.93E-04 | MDD | 2.56 | 6.63E-03 |
| Bilophila | Bilophila_wadsworthia | 9.18E-04 | 2.96E-04 | 2.24E-03 | 5.68E-04 | MDD | 2.81 | 1.67E-04 |
| Blautia | Blautia_obeum | 1.34E-02 | 1.68E-03 | 7.41E-03 | 8.37E-04 | HCs | 3.51 | 3.43E-04 |
| Blautia | Blautia_sp._CAG:237 | 3.67E-03 | 2.25E-03 | 6.04E-04 | 1.00E-04 | HCs | 3.15 | 2.53E-03 |
| Blautia | Blautia_sp._GD8 | 2.41E-03 | 3.71E-04 | 1.29E-03 | 1.68E-04 | HCs | 2.79 | 4.32E-03 |
| Blautia | Blautia_sp._KLE_1732 | 2.42E-03 | 3.81E-04 | 1.39E-03 | 1.89E-04 | HCs | 2.77 | 2.79E-02 |
| Blautia | Blautia_sp._Marseille-P2398 | 2.76E-03 | 6.02E-04 | 1.30E-03 | 1.59E-04 | HCs | 2.93 | 4.25E-03 |
| Blautia | Blautia_wexlerae | 1.14E-02 | 2.41E-03 | 5.89E-03 | 6.59E-04 | HCs | 3.50 | 1.12E-02 |
| Citrobacter | Citrobacter_freundii | 2.20E-03 | 1.47E-03 | 1.74E-04 | 4.56E-05 | HCs | 3.10 | 1.89E-02 |
| Clostridium | Clostridium_dakarense | 1.44E-03 | 4.18E-04 | 4.89E-04 | 1.10E-04 | HCs | 2.70 | 3.64E-02 |
| Clostridium | Clostridium_sp._CAG:62 | 7.65E-04 | 5.18E-04 | 1.86E-04 | 4.87E-05 | HCs | 2.52 | 1.65E-02 |
| Clostridium | Clostridium_sp._CAG:75 | 9.08E-04 | 6.35E-04 | 2.20E-04 | 9.03E-05 | HCs | 2.61 | 2.09E-02 |
| Clostridium | Clostridium_sp._CAG:81 | 4.22E-04 | 5.10E-05 | 1.00E-03 | 1.81E-04 | MDD | 2.51 | 1.14E-03 |
| Coprobacillus | Coprobacillus_sp._CAG:235 | 1.62E-03 | 2.65E-04 | 3.54E-03 | 7.45E-04 | MDD | 2.91 | 4.45E-02 |
| Coprococcus | Coprococcus_eutactus | 3.71E-03 | 8.50E-04 | 1.05E-03 | 2.04E-04 | HCs | 3.18 | 7.96E-03 |
| Coprococcus | Coprococcus_sp._ART55/1 | 1.59E-03 | 4.20E-04 | 3.08E-04 | 1.00E-04 | HCs | 2.87 | 2.62E-03 |
| Coprococcus | Coprococcus_sp._CAG:131 | 1.10E-03 | 2.98E-04 | 1.91E-04 | 7.16E-05 | HCs | 2.72 | 2.06E-02 |
| Dialister | Dialister_invisus | 9.24E-04 | 3.58E-04 | 1.52E-04 | 9.10E-05 | HCs | 2.62 | 4.00E-02 |
| Dialister | Dialister_succinatiphilus | 2.19E-03 | 7.95E-04 | 7.27E-04 | 5.23E-04 | HCs | 2.83 | 1.48E-02 |
| Dorea | Dorea_sp._CAG:105 | 2.36E-03 | 7.51E-04 | 1.13E-03 | 4.92E-04 | HCs | 2.88 | 3.21E-04 |
| Enterobacter | Enterobacter_cloacae | 3.13E-03 | 1.60E-03 | 8.85E-04 | 4.57E-04 | HCs | 3.01 | 5.23E-03 |
| Enterobacter | Enterobacter_sp._GN02315 | 6.16E-04 | 6.07E-04 | 1.38E-05 | 1.28E-05 | HCs | 2.51 | 2.90E-05 |
| Enterococcus | Enterococcus_faecalis | 3.03E-03 | 2.59E-03 | 3.13E-04 | 2.53E-05 | HCs | 3.08 | 1.27E-02 |
| Eubacterium | Eubacterium_hallii_CAG:12 | 1.73E-03 | 2.94E-04 | 7.29E-04 | 1.66E-04 | HCs | 2.78 | 8.83E-04 |
| Eubacterium | Eubacterium_sp._CAG:115 | 1.11E-03 | 6.59E-04 | 6.18E-05 | 2.37E-05 | HCs | 2.71 | 4.00E-02 |
| Eubacterium | Eubacterium_sp._CAG:146 | 1.98E-03 | 3.15E-04 | 3.58E-03 | 1.03E-03 | MDD | 2.99 | 2.53E-02 |
| Eubacterium | Eubacterium_sp._CAG:180 | 1.04E-02 | 3.04E-03 | 9.14E-03 | 4.65E-03 | HCs | 3.39 | 3.20E-03 |
| Eubacterium | Eubacterium_sp._CAG:202 | 1.10E-02 | 3.86E-03 | 3.68E-03 | 2.02E-03 | HCs | 3.50 | 3.04E-02 |
| Eubacterium | Eubacterium_sp._CAG:251 | 2.67E-03 | 8.16E-04 | 7.88E-04 | 3.69E-04 | HCs | 2.99 | 3.04E-02 |
| Faecalibacterium | Faecalibacterium_sp._CAG:74 | 3.72E-03 | 1.41E-03 | 5.23E-04 | 1.21E-04 | HCs | 3.26 | 9.53E-03 |
| Faecalibacterium | Faecalibacterium_sp._CAG:82 | 2.37E-03 | 2.05E-04 | 1.79E-03 | 2.14E-04 | HCs | 2.51 | 2.46E-02 |
| unclassified_p__Firmicutes | Firmicutes_bacterium_CAG:227 | 1.99E-03 | 5.83E-04 | 7.49E-04 | 1.41E-04 | HCs | 2.83 | 4.56E-03 |
| unclassified_p__Firmicutes | Firmicutes_bacterium_CAG:341 | 3.38E-03 | 1.48E-03 | 6.75E-04 | 3.93E-04 | HCs | 3.17 | 2.79E-02 |
| Klebsiella | Klebsiella_pneumoniae | 1.38E-02 | 5.25E-03 | 6.72E-03 | 2.84E-03 | HCs | 3.60 | 5.80E-03 |
| Lactococcus | Lactococcus_garvieae | 8.52E-04 | 5.96E-04 | 1.99E-04 | 1.65E-04 | HCs | 2.58 | 5.21E-04 |
| Parabacteroides | Parabacteroides_distasonis | 1.47E-03 | 2.11E-04 | 3.37E-03 | 4.14E-04 | MDD | 2.98 | 6.88E-05 |
| Parabacteroides | Parabacteroides_merdae | 2.52E-03 | 4.89E-04 | 3.99E-03 | 6.55E-04 | MDD | 2.86 | 1.89E-02 |
| Parasutterella | Parasutterella_excrementihominis | 5.64E-04 | 1.67E-04 | 1.27E-03 | 2.66E-04 | MDD | 2.61 | 6.42E-03 |
| unclassified_f__Peptostreptococcaceae | Peptostreptococcaceae_bacterium_VA2 | 1.01E-03 | 2.89E-04 | 3.46E-04 | 7.78E-05 | HCs | 2.55 | 3.30E-02 |
| Phascolarctobacterium | Phascolarctobacterium_sp._CAG:207 | 1.87E-03 | 5.18E-04 | 6.33E-03 | 1.27E-03 | MDD | 3.33 | 5.84E-05 |
| unclassified_p__Proteobacteria | Proteobacteria_bacterium_CAG:139 | 7.96E-04 | 3.42E-04 | 1.58E-03 | 3.49E-04 | MDD | 2.68 | 1.57E-02 |
| Ruminococcus | Ruminococcus_sp._5_1_39BFAA | 2.98E-03 | 6.60E-04 | 1.37E-03 | 1.69E-04 | HCs | 2.97 | 3.82E-03 |
| Ruminococcus | Ruminococcus_sp._CAG:17 | 2.04E-03 | 6.47E-04 | 8.85E-04 | 3.60E-04 | HCs | 2.77 | 1.19E-03 |
| Ruminococcus | Ruminococcus_sp._CAG:9 | 1.88E-03 | 4.43E-04 | 8.08E-04 | 1.09E-04 | HCs | 2.79 | 2.02E-03 |
| Ruminococcus | Ruminococcus_sp._JC304 | 1.69E-03 | 2.42E-04 | 9.70E-04 | 1.21E-04 | HCs | 2.62 | 1.74E-03 |
| Salmonella | Salmonella_enterica | 1.16E-03 | 3.15E-04 | 4.59E-04 | 1.16E-04 | HCs | 2.58 | 4.11E-02 |
| Subdoligranulum | Subdoligranulum_variabile | 2.47E-02 | 3.03E-03 | 1.43E-02 | 2.64E-03 | HCs | 3.69 | 4.48E-03 |
| Sutterella | Sutterella_wadsworthensis | 2.91E-04 | 9.59E-05 | 1.21E-03 | 3.21E-04 | MDD | 2.66 | 4.80E-03 |
