# Supplementary material for: Changes of gut microbiota reflect the severity of major depressive disorder: a cross sectional study
Source: Transl Psychiatry. 2023 Apr 28;13:137. doi: 10.1038/s41398-023-02436-z (PMC10147706; doi:10.1038/s41398-023-02436-z)
Supplement: Supplementary file 2 — Supplementary materials [file 41398_2023_2436_MOESM2_ESM.docx]

**SUPPLEMENTARY MATERIALS**

**Supplementary Tables**

Supplementary Table 1. Demographic information of the subjects

Supplementary Table 2. Discriminatory bacteria species between HCs and mild groups

Supplementary Table 3. Discriminatory bacteria species between HCs and moderate groups

Supplementary Table 4. Discriminatory bacteria species between HCs and severe groups

Supplementary Table 5. Discriminatory KOs between HCs and moderate groups

Supplementary Table 6. Discriminatory KOs between HCs and severe groups

Supplementary Table 7. Potential biomarker for identifying different severity of MDD

Supplementary Table 8. Discriminatory bacteria between HCs and MDD in female samples

Supplementary Table 9. Discriminatory bacteria between HCs and MDD in male samples

**Supplementary Figures**

**Supplementary Figure 1. Constitution of gut microbiota in HCs and MDD subgroups. A-B Top 10 bacteria of HCs and MDD subgroups.** The community bar plot illustrating the top 10 bacteria of HCs in relative abundance were different from 3 MDD subgroups. At both family (**A**) and genus (**B**) levels, the 3 MDD subgroups were all characterized by *Bacteroidaceae/Bacteroides* enrichment. **C enterotypes of HCs and MDD subgroups.** The major enterotype of HCs was *Faecalibacterium* while that were *Bacteroides* and *Bacteroides 2* in MDD subgroups.

**Supplementary Figure 2. Gut microbial characteristics among MDD subgroups.** At the species level, PCoA analysis showed that the 3 MDD subgroups cannot be separated from each other.

**Supplementary Figure 3. Associations of altered gut microbial species with KOs. A** correlation-based heatmap of differentially enriched KOs and species that correlated with either KOs. **B** correlation-based heatmap of differentially enriched KOs and species that highly correlated with either KOs (r^2^ > 0.25).

**Supplementary Figure 4 The α-diversity analysis between HCs and MDD of different genders at genus level.** The violin plots displayed similar trends of the changes between HCs and MDD in female (**A**) and male (**B**) groups in α-diversity analysis. The Dominance_D index increased while Simpson_1-D index and Shannon_H index decreased in MDD (Kruskal-Wallis test; *, *p*<0.05; **, *p*<0.01).

**Supplementary Figure 5 The PCoA analysis between HCs and MDD of different genders.** PCoA analysis was used to explore the general characteristics difference between HCs and MDD in female and male groups, PERMANOVA test was performed to quantify the statistical difference. HCs was significantly apart from MDD in both female and male groups (female, *p* = 0.0017; male, *p* = 0.0003).

**Supplementary Figure 6 Constitution of gut microbiota in different genders.** Bar plot was composed of the proportion of the top 10 bacteria in each subgroup, showed the composition of gut microbiota at the family level (**A**) and genus level (**B**) in each subgroup. At the family level, *Bacteroidaceae* and *Prevotellaceae* were enriched in both MDD-female and MDD-male groups, while at the genus level, *Bacteroides* and *Prevotella* were enriched. At the family level, HCs-female and MDD-female showed a higher proportion of *Bacteroidaceae* than that of the male subgroups, and a lower proportion of *Enterobacteriaceae* and *Prevotellaceae* than that of the male subgroups.

**Supplementary Figure 7 Differentially enriched bacteria species of HCs and the 3 MDD subgroups.** LEfSe analysis was performed to identify the differential enriched bacteria between HCs and MDD in female (**A**) and male (**B**) samples. The error bars indicated standard error of mean. The left bar plot represented HCs, the right ones represented MDD. (**C**) Venn diagram of results of LEfSe analysis. The female group and the male group shared 22 species of bacteria, and the number of shared bacteria accounted for a larger proportion in each gender group.

**Supplementary Figure 8 The co-occurrence network of differentially enriched bacteria between HCs and MDD in different genders.** Based on the metagenome abundance data (RPKM), we constructed co- occurrence networks of differentially enriched bacteria between HCs and MDD in different genders (SparCC, *p*<0.05, r^2^>0.25). (**A**) The co-occurrence network of differentially enriched bacteria in female samples. *Bacteroides* was significantly enriched while *Eubacterium* and *Blautia* was remarkably depleted in MDD. The enriched bacteria were negatively associated with those depleted ones. (**B**) The co-occurrence network of differentially enriched bacteria in male samples. MDD in the male samples was also characterized by enrichment of *Bacteroides*. But the depleted bacteria were much more than the female sample, including *Eubacterium*, *Blautia*, *Ruminococcus* and *Coprocooccus*. The enrichment changes of the three species of *Eubacterium* were not consistent, *Eubacterium_sp._CAG:146* was enriched in MDD, *Eubacterium_hallii* and *Eubacterium_hallii_CAG:12* was depleted in MDD.
