# Supplementary figures and images for: Changes of gut microbiota reflect the severity of major depressive disorder: a cross sectional study
Source: Transl Psychiatry. 2023 Apr 28;13:137. doi: 10.1038/s41398-023-02436-z (PMC10147706; doi:10.1038/s41398-023-02436-z)

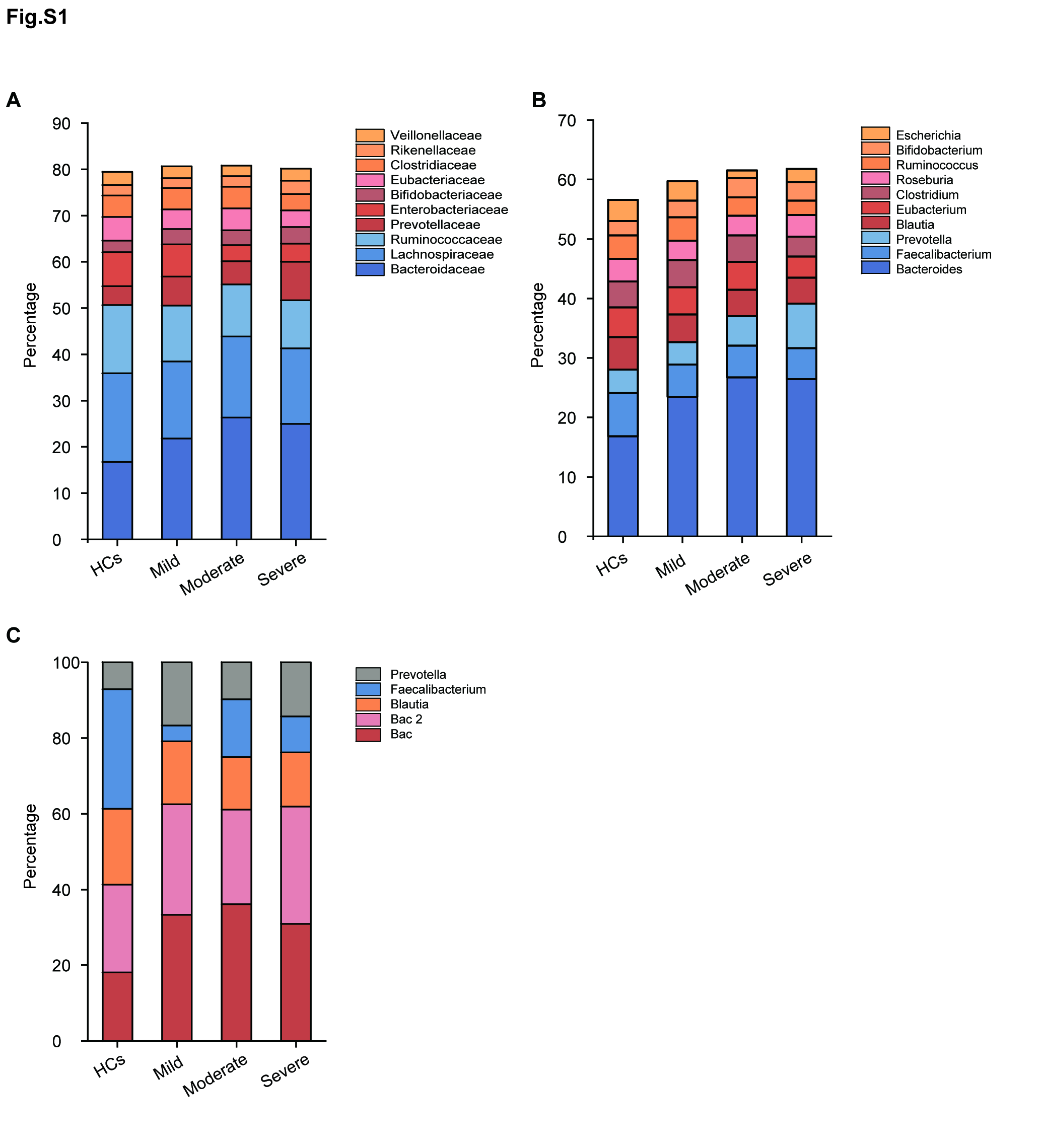

Supplement: Supplementary file 5 — supplementary figure 1 [file 41398_2023_2436_MOESM5_ESM.tif]

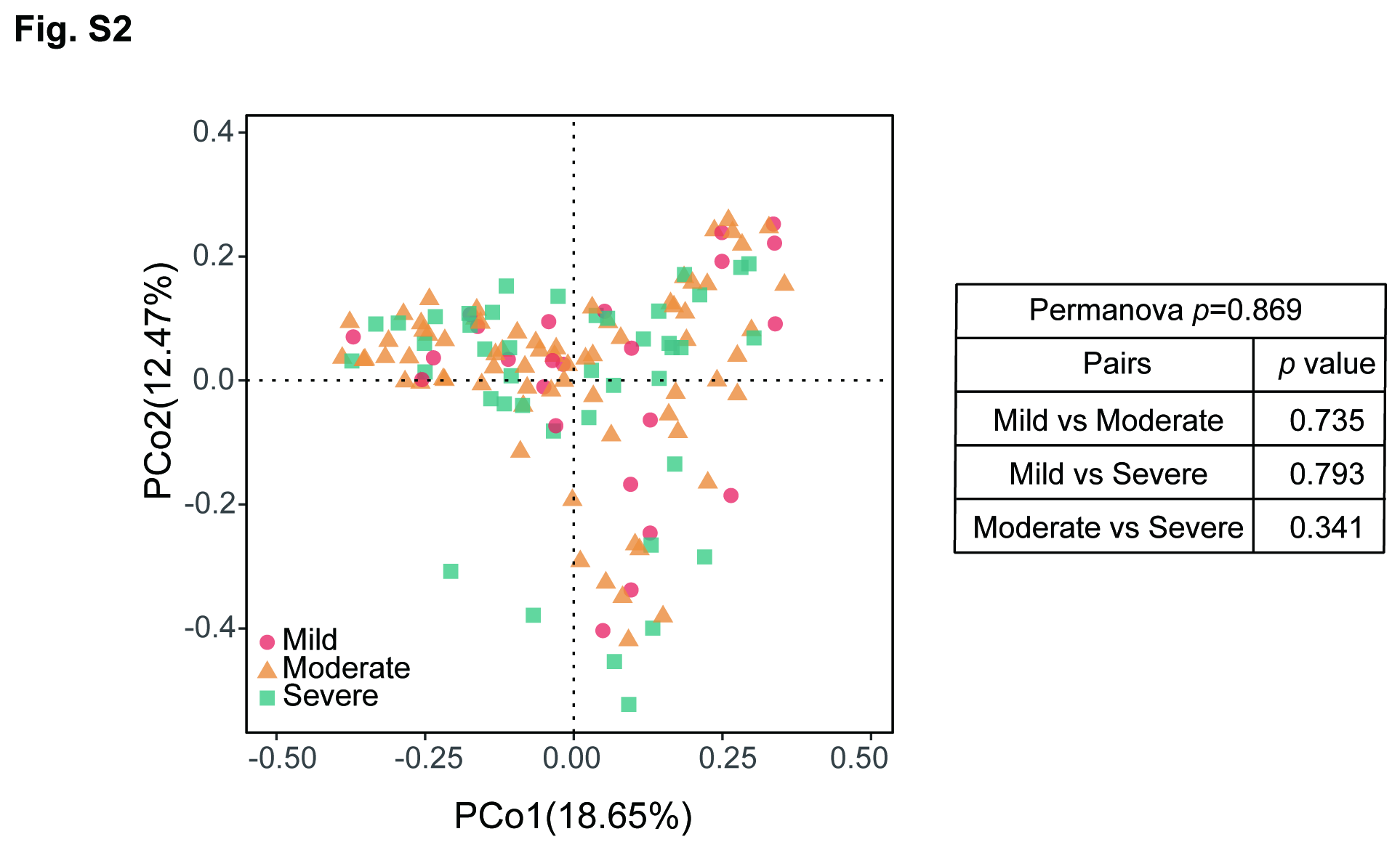

Supplement: Supplementary file 6 — supplementary figure 2 [file 41398_2023_2436_MOESM6_ESM.tif]

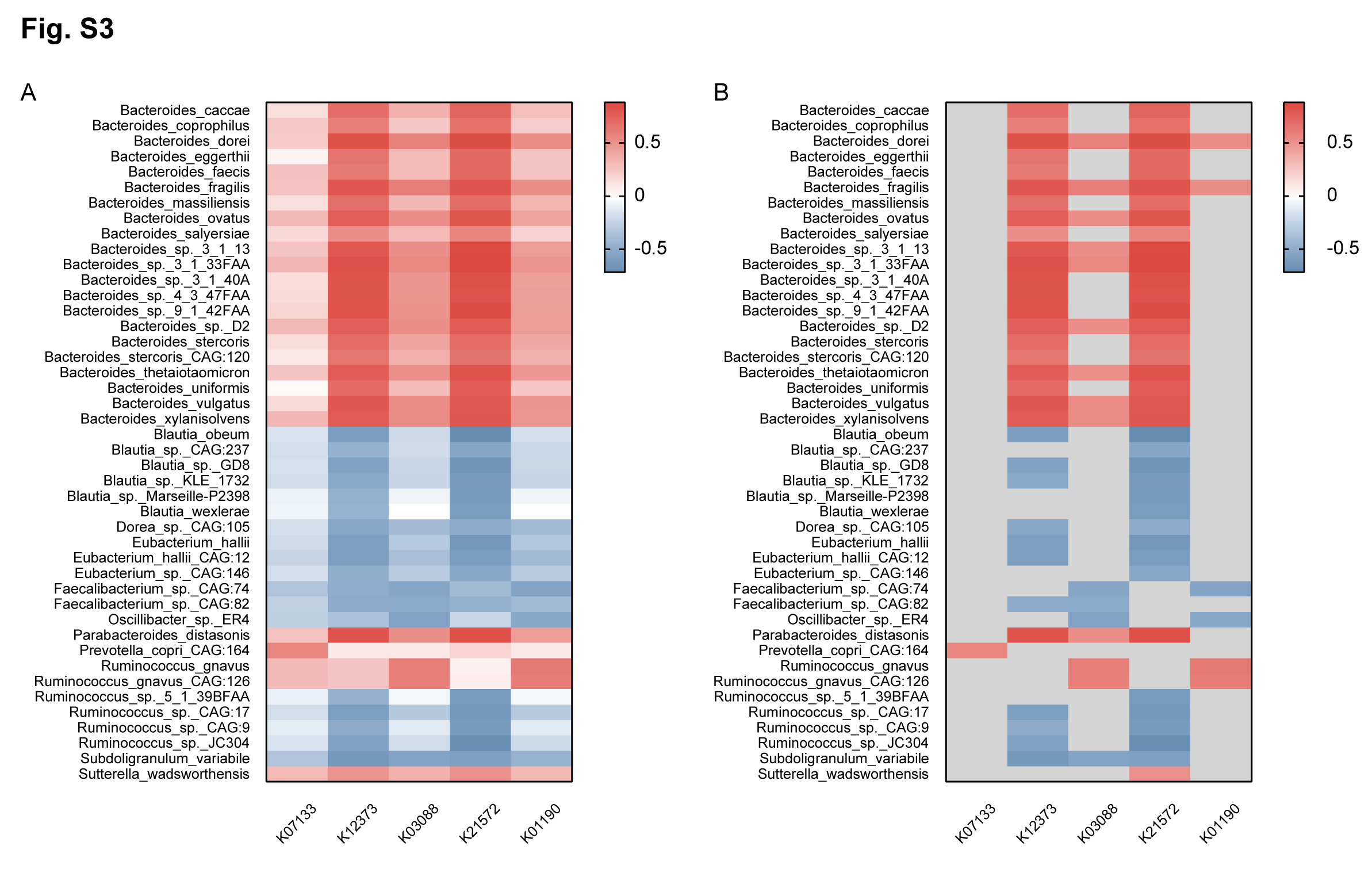

Supplement: Supplementary file 7 — supplementary figure 3 [file 41398_2023_2436_MOESM7_ESM.tif]

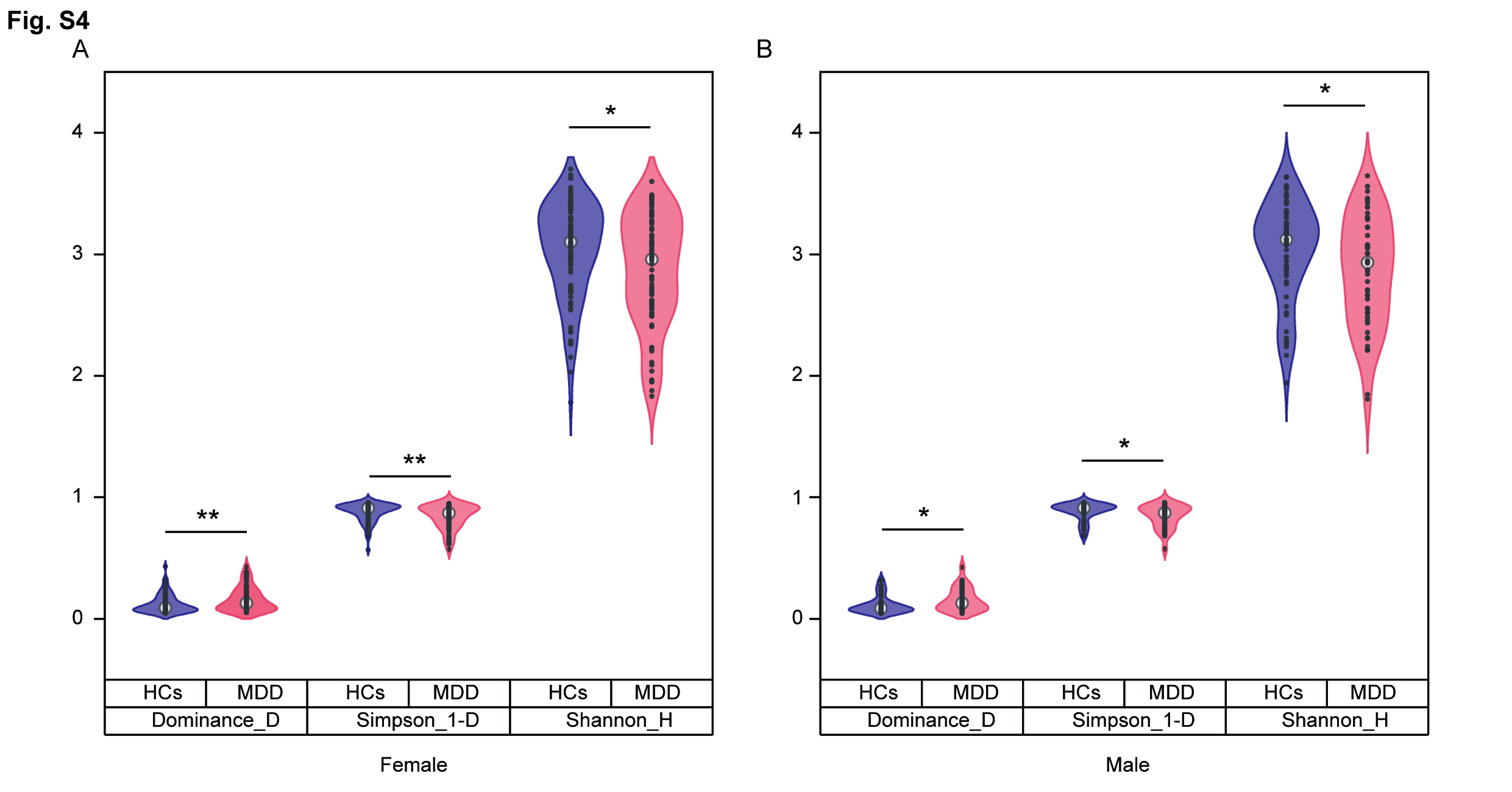

Supplement: Supplementary file 8 — supplementary figure 4 [file 41398_2023_2436_MOESM8_ESM.tif]

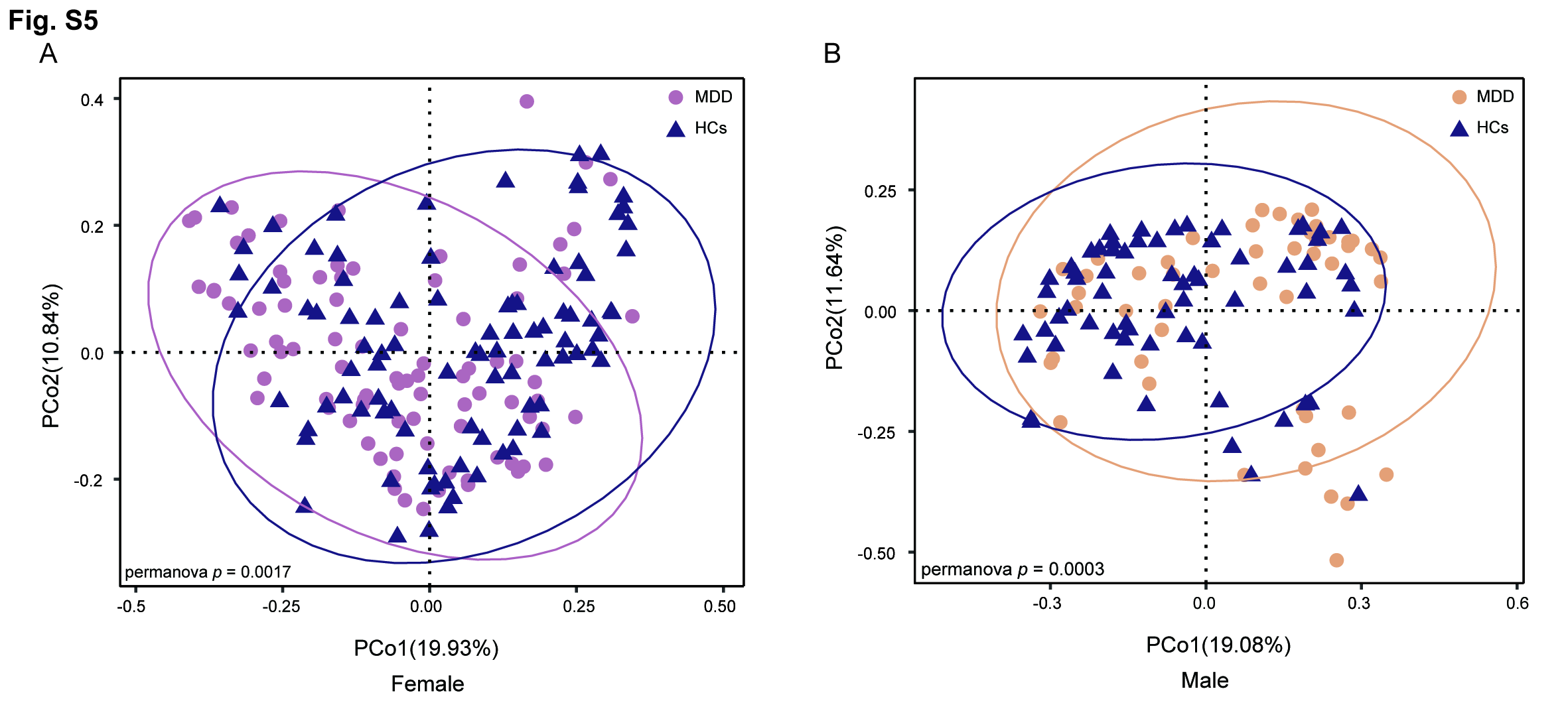

Supplement: Supplementary file 9 — supplementary figure 5 [file 41398_2023_2436_MOESM9_ESM.tif]

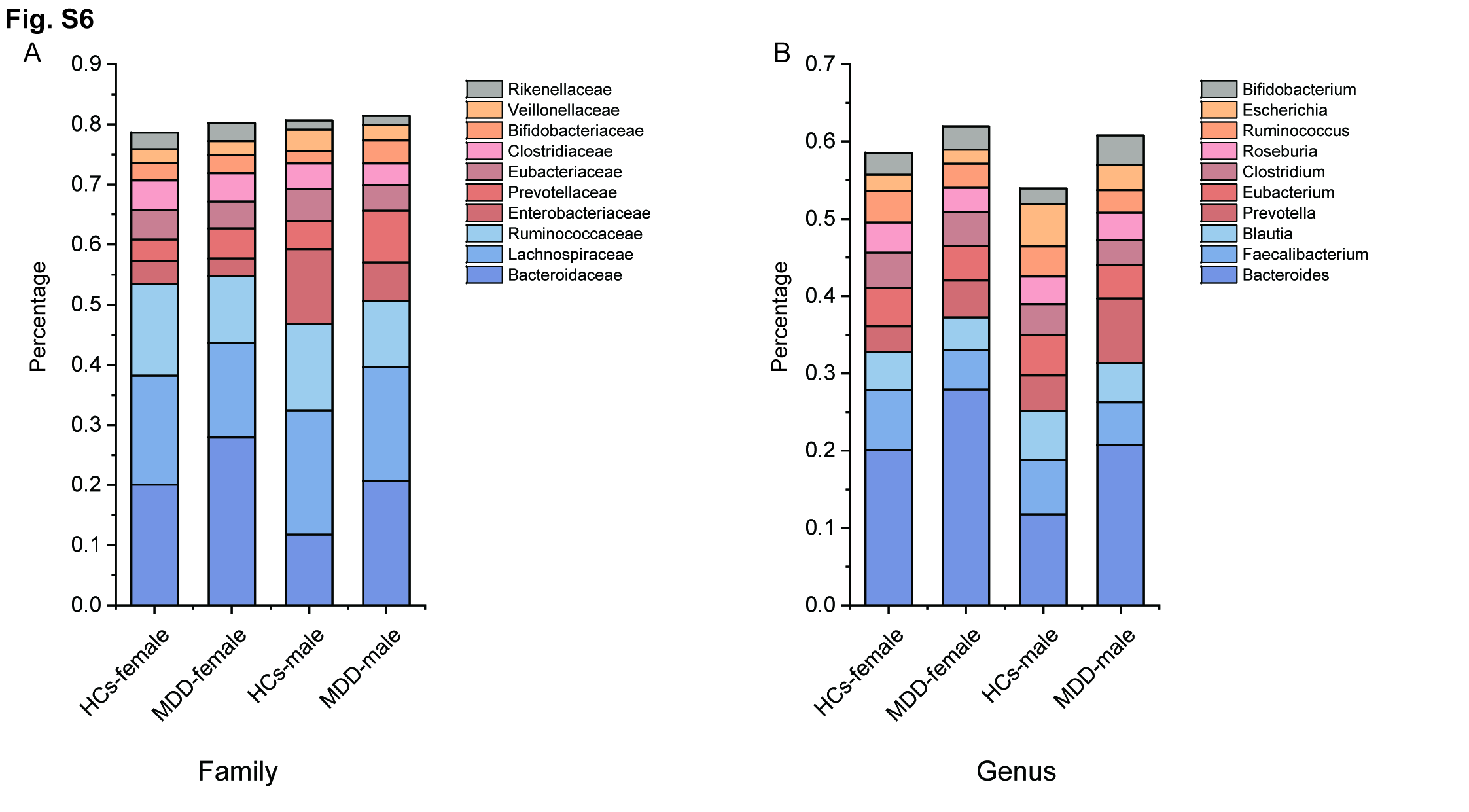

Supplement: Supplementary file 10 — supplementary figure 6 [file 41398_2023_2436_MOESM10_ESM.tif]

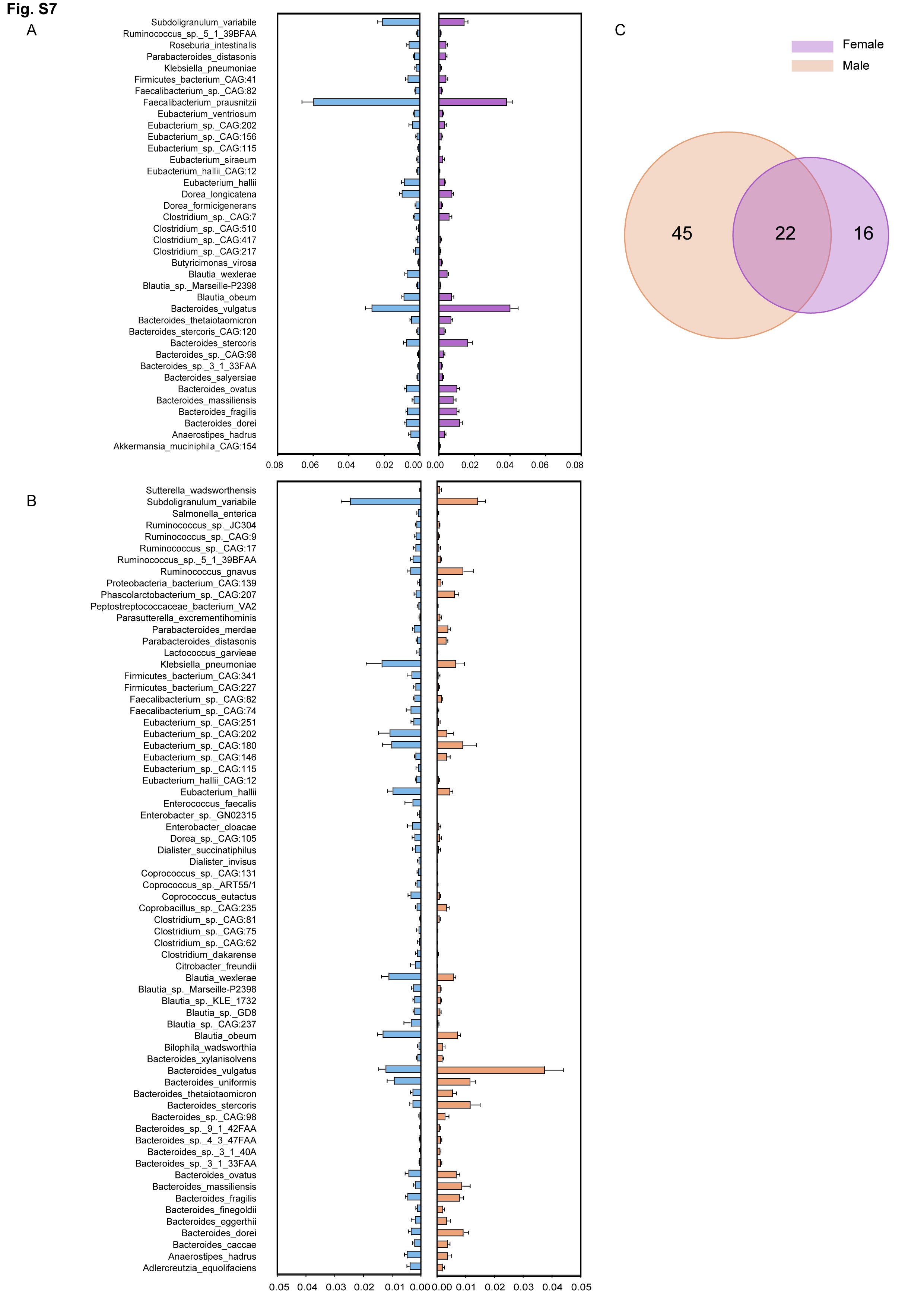

Supplement: Supplementary file 11 — supplementary figure 7 [file 41398_2023_2436_MOESM11_ESM.tif]

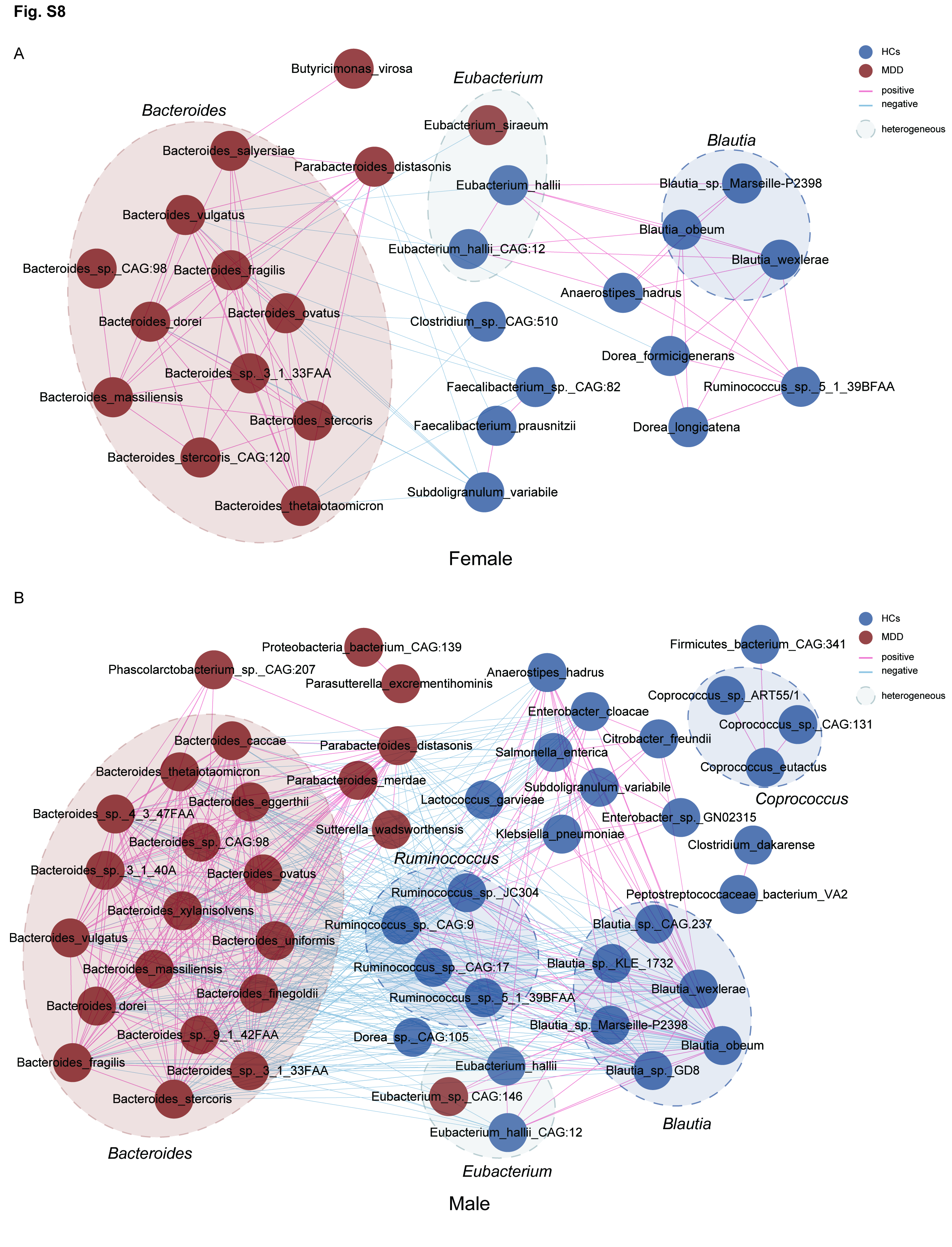

Supplement: Supplementary file 12 — supplementary figure 8 [file 41398_2023_2436_MOESM12_ESM.tif]
